# Advances in Use of Nanomaterials for Musculoskeletal Regeneration

**DOI:** 10.3390/pharmaceutics13121994

**Published:** 2021-11-24

**Authors:** Josef Jampilek, Daniela Placha

**Affiliations:** 1 Department of Analytical Chemistry, Faculty of Natural Sciences, Comenius University, Ilkovicova 6, 842 15 Bratislava, Slovakia; 2 Nanotechnology Centre, CEET, VSB-Technical University of Ostrava, 17. Listopadu 2172/15, 708 33 Ostrava-Poruba, Czech Republic; 3 Centre ENET, CEET, VSB-Technical University of Ostrava, 17. Listopadu 2172/15, 708 33 Ostrava-Poruba, Czech Republic

**Keywords:** nanomaterials, nanocomposites, cartilage, bones, implants, healing, musculoskeletal disorders

## Abstract

Since the worldwide incidence of bone disorders and cartilage damage has been increasing and traditional therapy has reached its limits, nanomaterials can provide a new strategy in the regeneration of bones and cartilage. The nanoscale modifies the properties of materials, and many of the recently prepared nanocomposites can be used in tissue engineering as scaffolds for the development of biomimetic materials involved in the repair and healing of damaged tissues and organs. In addition, some nanomaterials represent a noteworthy alternative for treatment and alleviating inflammation or infections caused by microbial pathogens. On the other hand, some nanomaterials induce inflammation processes, especially by the generation of reactive oxygen species. Therefore, it is necessary to know and understand their effects in living systems and use surface modifications to prevent these negative effects. This contribution is focused on nanostructured scaffolds, providing a closer structural support approximation to native tissue architecture for cells and regulating cell proliferation, differentiation, and migration, which results in cartilage and bone healing and regeneration.

## 1. Introduction

Degenerative diseases of the bones and joints affect millions of people. Fractures of the hands, hips, and spine caused by osteoporosis are associated with significant morbidity and mortality. Destruction and deformity of the joints and other complications caused by arthritis not only make movement difficult, but reduce the ability to perform routine activities, which results in an overall reduced quality of life for patients, among other things. Prolonged life has affected many aspects of the everyday life of older people, one of which is the difficulty of movement common in older people who suffer from problems such as fear of falling (81.7%), inability to stand without arm support (81.1%), inability to climb up the stairs (81.3%), and slow walking speeds (71.7%), so they prefer not to leave the flats (50%) [1,2,3].

**Figure 1:**
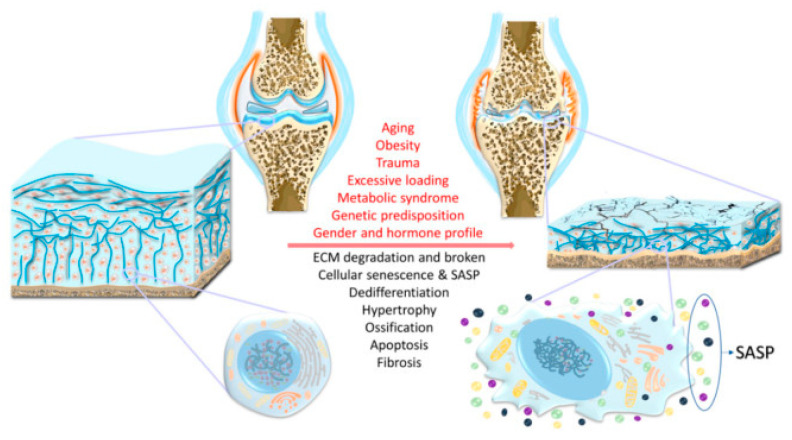
Risk factors (described in red) contributing to the development of osteoarthritis (OA). Description of structural alteration and chondrocyte-specific changes in osteoarthritis (described in black). ECM: Extracellular matrix; SASP: Senescence-associated secretory phenotype. Adapted from [12], MDPI, 2017.

Osteoporosis and arthritis are among the most common and serious diseases of the musculoskeletal system [4,5,6]. Osteoporosis is a disease in which bone weakness increases the risk of fractures and is the most common cause of fractures (vertebrae, forearms, and hip bones) in the elderly, especially women. Osteoporosis can also occur as a result of a number of diseases (alcoholism, anorexia, hyperthyroidism, kidney disease, and surgical removal of the ovaries) or treatment (antihypertensives, chemotherapy, proton pump inhibitors, selective serotonin reuptake inhibitors, and glucocorticoids). It is currently estimated that more than 200 million people suffer from osteoporosis [2,4,7,8]. In turn, arthritis is an inflammatory disease of the joints. It is manifested by swelling, redness, pain, and restriction of movement. There are several types of arthritis, the most common of which are rheumatoid arthritis (RA) and osteoarthritis (OA) [5,6,9]. OA is a degenerative disease of the joints in which the articular cartilage and the bones beneath it break down. It is the most common form of arthritis, affecting about 3.3% of the world’s population. The symptoms progress slowly over the years, but only the joints are affected. The real causes are unknown [9,10,11,12,13]. A description of the structural alteration of cartilage and chondrocyte-specific changes in OA with the indicated risk factors is shown in Figure 1 [12]. The risk factors that can contribute to the development of OA are named in Figure 1; however, they are not the only factors. In this figure, a scheme of cartilage structural alteration and chondrocyte-specific changes in OA is also depicted including cartilage breakdown, subchondral bone thickening, formation of osteophytes and corpus liberum, narrowed joint space, thickened and fibrotic ligaments, and joint capsule hypertrophy. A decrease in chondrocyte numbers in cartilage is seen by increased apoptosis. Chondrocytes may be subject to dedifferentiation and form the hypertrophic and senescent phenotypes. Senescence-associated secretory phenotype (SASP) is synthesized and secreted by chondrocytes creating a detrimental environment within the joint [12]. While the incidence of OA is much higher than that of RA (0.1–2.0% of the world’s population), the latter is a far more complex disease having over 50 forms with an overriding and variable influence of inflammation and immune mediated cellular effects in all of these. RA is a systemic inflammatory autoimmune disease that leads to chronic inflammation of the synovial tissue, causing irreversible damage to cartilage and joint bones. However, inflammation can also affect the eyes, heart, and lungs, leading to cardiovascular and respiratory diseases. Thus, in addition to a radical reduction in quality of life, RA is associated with increased mortality. The causes of RA are unknown. Risk factors include genetic predisposition, excessive smoking, alcoholism, obesity, and environmental exposure to asbestos [5,14,15,16]. An important distinction between OA and RA is that OA is mechanically driven with a minor inflammatory component compared to RA; hence, OA predominantly affects the major weight bearing joints such as the hip and knee, while RA, which is predominantly driven by inflammation and immune processes, also affects the smaller joints and is a symmetrical disease (i.e., with the same interphalangeal and metacarpal phalangeal joints of the fingers on both hands affected). This is a specific feature of RA not seen in OA. Furthermore, the ankle joints are rarely affected by OA, while they can be affected by RA [5,6,16].

Many different treatment approaches are being developed for the burning problem of increasingly common musculoskeletal degenerative diseases. Treatment options of the musculoskeletal system are non-pharmacological, pharmacological, and surgical.

Efforts to prevent fractures in people with osteoporosis include diet, exercise, fall prevention, and lifestyle changes (reducing smoking and alcohol). Standard treatment is hormone replacement therapy (HRT: estrogen + progestogen, tibolone, raloxifene, testosterone, calcitonin), bisphosphonates (risedronate, etidronate, alendronate), teriparatide (recombinant parathyroid hormone), strontium ranelate, denosumab, and vitamin D supplementation [4,7,8,17,18,19,20].

Arthritides are incurable diseases for this moment, so the goal of treatment is to achieve remission or a low activity of the disease. The later the treatment is started, the worse the results and the irreversible damage to the joints are. Non-pharmacological treatment is based on regular exercise (weight reduction, physical activity), rehabilitation, and manipulation therapy to strengthen muscles and maintain maximum mobility and joint functionality. In advanced stages of the disease, some damaged joints can be surgically removed and replaced with artificial implants (joint endoprostheses). Surgical treatment of the patient also relieves pain in the affected joint. Pharmacological treatment includes two basic groups of drugs, which are usually combined: drugs that reduce inflammation and pain, and drugs that reduce the progression of structural damage (i.e., inhibit the destruction of articular cartilage and induce the balance of its metabolism). Non-steroidal anti-inflammatory drugs (naproxen, ibuprofen, and COX-2 selective inhibitors), paracetamol, and weak opiates (tramadol) are used to reduce inflammation and pain. In the case of acute inflammation, glucocorticoids (hydrocortisone) can be given. Disease-modifying antirheumatic drugs reduce the progression of structural damage. They can be divided into synthetic and biological drugs. Synthetic drugs are further divided into conventional synthetics (methotrexate, sulfasalazine, leflunomide, hydroxychloroquine, chloroquine, and gold salts) and targeted synthetic JAK kinase inhibitors (baricitinib, tofacitinib). Biologicals are antibodies (e.g., adalimumab, etanercept, infliximab, anakinra, tocilizumab, abatacept, rituximab) against pro-inflammatory mediators/agents of humoral and cell-mediated immunity [5,16,21,22,23,24,25,26]. In this context, it is necessary to mention that there are also many dietary supplements on the market that are intended to prevent or alleviate diseases of the musculoskeletal system. Agents that inhibit the destruction of articular cartilage are so-called chondroprotectives; currently recommended are glucosamine sulfate and chondroitin sulfate (not to be chloride salts), hyaluronic acid, avocado-soybean unsaponifiables, diacerein, *Boswellia serrata* extract, curcumin, S-adenosyl methionine, methylsulfonylmethane, and rose hip. Alternatively, fish liver oil, omega-3 fatty acids, vitamins A, C, and E in combination, vitamin K, vitamin D, ginger, Russian olive (*Elaeagnus angustifolia*), nettle (*Urtica dioica*), and collagen/gelatin are listed as beneficial dietary supplements [23,25,26,27,28,29,30].

In addition to various types of drugs including biological treatments, modern materials made by nanotechnology have begun to be used. These are nanosystems for drug delivery with targeted distribution and modified drug release, nanodiagnostics, and various materials with their own antimicrobial activity [31,32,33,34,35,36,37,38]. In this context, it is necessary to mention that nanosupplements for osteoporosis management and for the supply of vitamins and many other biologically active agents can be found in the development and on the market [30,39,40,41]. It is mainly nanosized calcium in tablets or nanopowdered eggshells, which is added to yogurt or milk, resulting in high-calcium yogurt, calcium-fortified milk. Calcium treated in this way has considerably increased bioavailability and effectively serves as a supportive treatment for diseases related to calcium deficiency in the bones. Additionally, vitamins and many other biologically active agents are reformulated into nanosystems with increased bioavailability and stability, especially with increased distribution/targeted delivery to bone or cartilage tissue [31,33,34,39,40,41,42].

Another application of nanomaterials, nanocomposites (NCPs), can be found in tissue engineering, where they began to be used as scaffolds for the development of biomimetic materials involved in the repair and healing of damaged tissues and organs [43,44,45,46,47]. The term “tissue engineering” was proposed as early as 1987 and is defined by the National Science Foundation as “…the application of principles and methods of engineering and life sciences toward fundamental understanding the structure–function relationship between normal and mammalian tissues and development of biological substitutes to restore, maintain, and improve tissue functions.” [48]. NCPs replace damaged pieces of tissue and, in this case, are designed to facilitate the growth of bone and cartilage cells. It is therefore an innovative strategy to further help patients affected by the above-mentioned diseases, which radically reduce mobility and quality of life. These NCPs developed as replacements for bones, joints, and cartilage can be supplemented with other substances/drugs that have local antimicrobial, anti-inflammatory, or even anti-cancer action in order to maximize the regenerative effects for tissues associated with the musculoskeletal system, which is pathologically altered.

This contribution is focused on nanostructured scaffolds providing a structural support approximation to native tissue architecture for cells and regulating cell proliferation, differentiation, and migration, which results in cartilage and bone healing and regeneration.

## 2. Applied Nanomaterials

Various materials, cells, and bioactive compounds are studied and assembled in tissue engineering to provide a three-dimensional (3D) scaffold that would be used to induce and/or stimulate differentiation signals and thus promote regeneration of damaged tissue. The 3D structure, ideally made of biomimetic materials, is populated by cells and must therefore provide a suitable environment for cell growth, proliferation, and differentiation. Stem cells, undifferentiated cells with the ability to divide and give rise to various forms of specialized cells, are installed in the 3D scaffold. Frequently, the scaffold contains a variety of growth factors to direct the behavior of the cells to the desired process, where the ultimate goal is to create a fully functional organ/tissue. In order for a material to be used in tissue engineering, it needs to meet basic requirements such as (i) biocompatibility; (ii) biointegration; (iii) mechanical stability; (iv) easy production and handling; and (v) low production costs [49,50,51]. A nice example is the use of materials based on chondroitin sulfate, hyaluronan, or collagen-binding peptides for bioscaffolds. All these structural motifs contribute to the proliferation and differentiation of stem cells in the scaffold environment and are thus able to cause tissue regeneration and healing [52,53].

Nanomaterials seem to be the ideal nanostructured scaffold in tissue engineering that aims to replace/repair damaged tissues in the human body. Nanotechnologies are undoubtedly one of the key technologies of the new millennium. This field is constantly growing and provides/specializes in the development of materials with unique dimensions and thus unique properties that have enabled significant breakthroughs in many fields of human activity and have entered medicine and biological engineering. Nanoscale materials change their physical and chemical properties [54,55,56]; in this way, a number of industrial, pharmaceutical, and medical products have been improved and innovated [57,58,59,60,61,62,63,64,65,66,67] including various biocompatible NCPs for the construction of medical implants. The success of all these innovative biomedical applications is reflected in the size of the international nanomedicine market, which is estimated at $293.1 billion in 2022 and growing to $350.8 billion in 2025. On the other hand, there are barriers to their full use, especially their toxicological problems [50,68,69].

A variety of biocompatible materials are used to create scaffolds. Such a scaffold is a template for cell adhesion, differentiation, proliferation, and regeneration/growth, which means that the scaffold must have a suitable microenvironment for growing cells. This can be ensured by suitable surface modification/functionalization of the chemical structure to provide minimal cytotoxicity, high biocompatibility, and adhesion to the cells of the whole artificial scaffold. Therefore, it is not surprising that nanomaterials have already been used as surgical implants for tissue repair and regeneration in dentistry and orthopedics, the properties of NCPs to promote cartilage and bone growth being used in combination with their ability to anchor anti-inflammatory, anti-infective, or anti-cancer drugs [69,70,71,72,73,74].

**Figure 2:**
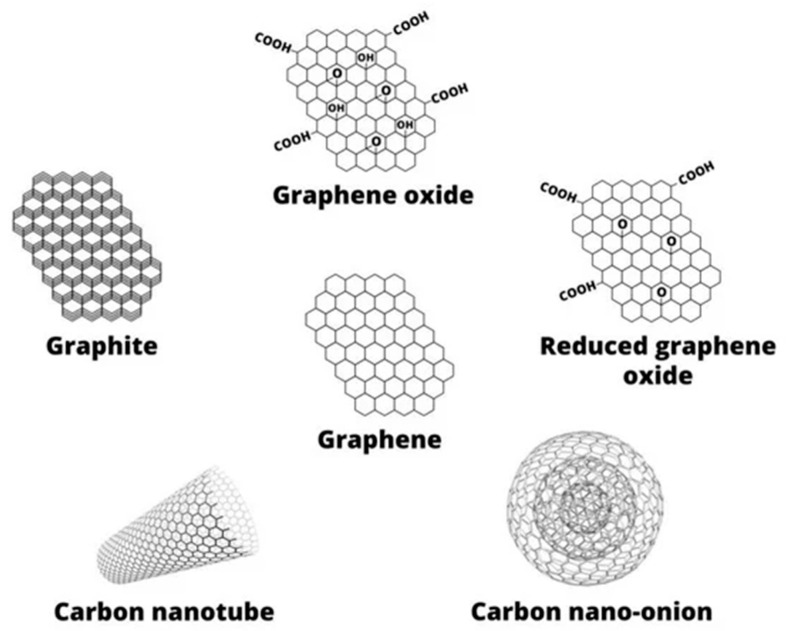
Graphene based materials. Graphene is a basic unit from which other forms of various shapes (nanotubes, onions, etc.) are derived. Graphene oxide and reduced graphene oxide bear oxygen-containing groups such as carboxylic groups (–COOH), hydroxylic groups (–OH), and ether groups (–O–). Graphite consists of individual graphene sheets. Adapted from [33], MDPI, 2019.

NCPs used as implants include organic–inorganic, inorganic–inorganic, and bioinorganic nanomaterials and are, in general, based on the following materials: (i) polymeric (e.g., poly(l-lactic acid) (PLLA), poly(d,l-lactic-co-glycolic acid) (PLGA), polymethyl methacrylate (PMMA), polyvinyl alcohol (PVA), chitosan (CS), alginate (ALG), gelatin (GLT), collagen (CLG) glucan, hyaluronic acid); (ii) carbon-based (graphene-based materials, carbon nanotubes (CNTs), carbon dots (CDs), graphene oxide (GO), etc., (see Figure 2); (iii) ceramic (hydroxyapatite (HA), aluminosilicates); and (iv) metal (including magnetic) [33,71,72,75,76,77,78].

Their synthesis/production varies depending on the starting material and applications and cannot be briefly described. In the next sections of this paper, where the individual materials are discussed, their preparation is described briefly.

## 3. Nanomaterials for Cartilage Healing and Regeneration

Cartilage is an important tissue providing the structure and function of support and protection in the human body. Its degeneration is induced if the catabolic factors are higher than the anabolic factors. In the case of damage, the regeneration ability is poor because of its hypocellular and hypovascular tissue, and it is difficult to repair. Treatment of its injury, degeneration, and defects presents a meaningful problem of clinical research because currently available treatments do not provide a perfectly compliant solution. Tissue engineering working with soft materials is a very promising way to repair damaged cartilage and bone tissue. It develops suitable substrates bearing the required physical, chemical, and biological stimuli for cell proliferation for direct chondrogenesis [79,80,81]. Nanotechnologies seem to be a strategic tool in how to diagnose, prognose, monitor, and/or clinically manage OA. Smart delivery drug systems, nanotubes, magnetic nanoparticles (NPs), NCPs, biological agents, and biomimetic regenerative platforms to support cell and gene therapies for stopping OA and promoting bone and cartilage repair have been described in many research studies. Nanomaterials and NCPs can be combined with various cell, gene, and biological therapies and form a new generation of future OA therapeutics. The physical and mechanical properties of the scaffolds can be enhanced using various methods such as incorporation of nano particles, cross-linking, and others [37].

Currently, there is no definitive treatment for articular cartilage defects developed. Treatment procedures often end with an artificial knee joint replacement [82]. Thus, articular cartilage regeneration is a challenge for research in orthopedics and tissue engineering. The following overview shows many ways in helping to repair and regenerate it.

**Figure 3:**
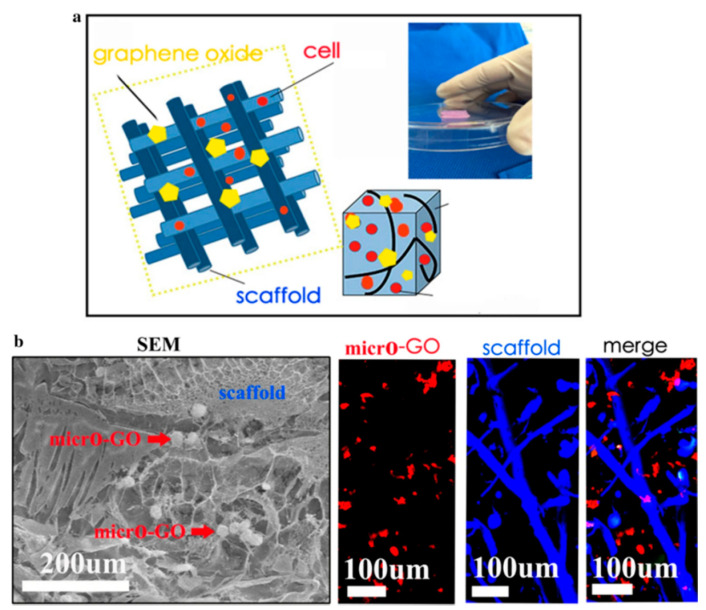
3D-printed scaffold containing graphene oxide (GO) for the cartilage layer construction. (**a**) Scheme of 3D-printed scaffold containing GO and chondrocytes (cells), (**b**) SEM and immunofluorescence in vitro evaluation of micro-GO presence in scaffold. The scaffold (in blue color) forms nets, in which micro-GO (in red color) and chondrocytes are localized. Micro-GO are flakes with length less than 100 µm. Toluidine blue and 1,1-dioctadecyl-3,3,3,3-tetram-ethylindocarbocyanine perchlorate (DiI; Sigma, St. Louis, MO, USA) were used to label micro-GO flakes within the scaffold. Adapted from [83], BMC, 2020.

A biological tissue can be accurately imitated using 3D printing. Its construction includes seed cell layers, biological activity factors, and biologically compatible scaffolds. GO in the amount of 10 wt% was successfully tested for the preparation of a 3D-printed scaffold with chondrocyte-proliferation potential (see Figure 3). The newly formed cartilage matrix extended along the scaffold and the border of the cartilage and matured as confirmed by scanning electron microscopy, immunofluorescence, and in vivo research. It was visible that the scaffold was entwined in a net. The micro-GO flakes with the size of less than 100 µm were localized inside the cross-linked scaffold structure. The cartilage growth on the 3D-printed GO scaffold was thicker than that on the 3D-printed scaffold without GO, which confirmed GO potential for a cartilage matrix [83].

Rajzer et al. [84] utilized a 3D printing process to produce a polycaprolactone (PCL)/graphene (GR) scaffolds with antimicrobial properties using short filament sticks. New filament materials with GR nanoplatelets in concentration of 0.5, 5, and 10 wt% were prepared using injection molding. The presence of GR enhanced the mechanical properties of filaments. The filaments were used in a commercial 3D printer to print scaffolds for nasal cartilage replacement, and the proliferation of chondrocytes was proven [84]. A GO-modified 3D acellular cartilage extracellular matrix (ACM) scaffold for cartilage repair was prepared by GO and ACM crosslinking using 1-ethyl-3-(3-dimethylaminopropyl)carbodiimide hydrochloride and *N*-hydroxy succinimide. GO addition enhanced the internal structure and mechanical properties of the scaffold. Cell adhesion, cell proliferation, and chondrogenic differentiation in vitro were promoted, and good biocompatibility and mild inflammatory response were proven with subcutaneous implantation in rats. After 12 weeks of implantation, the composite scaffold loaded with bone marrow mesenchymal stem cells (MSCs) completely bridged the cartilage defects in the rabbit knee with hyaline cartilage [85]. Sericin/reduced graphene oxide (rGO) NCPs with sericin/rGO ratios 10:1, 50:1, and 100:1 were studied in [86]. The NCPs promoting glycosaminoglycan and CLG levels can be promising in repairing articular cartilage in knee joints in nursing care. 3D bioprinted GO-doped GLT based scaffolds for promoting chondrogenic differentiation of human bone marrow MSCs were prepared using GO–GLT methacrylate (GLT-IMA)-poly(ethylene glycol)diacrylate (PEGDA) as a biocompatible photopolymerizable bioink. The structure of 3D printed scaffold was GLT–IMA–PEGDA–GO, and the scaffold increased the glycosaminoglycan and CLG levels after chondrogenic differentiation of hMSCs [87]. Bio-inks for 3D bio-printing of osteochondral scaffolds with different ratios of CS, GLT, and hyaluronic acid were prepared also containing GR with the ratio of 0.024, 0.06, and 0.1 wt% to improve the mechanical properties of the bioscaffold. To study the biocompatibility of the scaffolds, bone MSCs were inoculated onto the bioscaffolds. CS/GLT/hyaluronic acid scaffolds containing GR showed a good 3D porous structure; porosity was more than 80%; the mechanical strength was improved; pore walls were smoother and thicker; and bone MSCs successfully grew on the scaffolds [80].

PCL scaffolds with grid-like structure and periodic lattice containing GR nanoplatelets were prepared for cartilage tissue applications. The porous scaffold construction was conducted using a layer-by-layer assembly. The GR/PCL composite scaffolds showed good cytocompatibility and non-toxicity using mouse bone marrow MSCs that proliferated well on the scaffolds and confirmed a chondrogenic differentiation [88]. Using the plasma arc discharge method, Holmes et al. [79] prepared a carbon nanomaterial mixture containing GR nanoplatelets and single-walled carbon nanotubes. The mixture was added into electrospun PCL microfibrous scaffolds with or without poly-l-lysine surface coating. The scaffolds containing carbon nanomaterial showed highly enhanced mechanical properties and improved stem cell adhesion, proliferation, and chondrogenic differentiation, thus the material is promising for cartilage formation in clinical applications [79]. A macroporous polymeric scaffold of chitin and PCL was prepared by the lyophilization technique. Transforming growth factor-β (TGF-β) was encapsulated in chondroitin sulfate (ChS) NPs and incorporated in the chitin-PCL scaffold to study a prolonged TGF-β release. TGF-β-ChS NPs were characterized using a dynamic light scattering particle sizer and SEM, and it was proven that they were spherical particles of a 230 ± 20 nm. The composite scaffold was stable in swelling and degradation studies. The presence of TGF-β positively influenced the attachment and proliferation of rabbit adipose derived MSCs. In addition, an increased proteoglycan deposition was confirmed in the presence of TGF-β [81].

Umbilical cord MSCs loaded with GO granular lubricant were used to treat a knee OA animal model. Methods of treatment of 24 New Zealand rabbit models of knee OA were established. The models were divided into the blank group, the GO group, the umbilical cord MSCs group, and the GO + umbilical cord MSC group, each group including six animal models. The best results of NO, IL-6, TNF-α, GAG, and COL-II were obtained in the case of the GO + umbilical cord MSC group. Cartilage repair was confirmed in this group [89]. Shamekhi et al. [90] prepared scaffolds based on CS containing different amounts of exfoliated GO NPs (from 0 to 0.3 wt%). The physical and mechanical properties of the prepared samples were enhanced with the increasing GO content. The human articular chondrocytes were seeded on the scaffolds, and a higher proliferation was observed in samples with higher GO percentage [90]. A GO-doped electrospun PLGA nanofibrous membrane was prepared using the electrospinning technique, and in vitro cell assays were used for its evaluation using rabbit models. There was no change in the 3D microstructure of filament after GO mixing with PLGA. By means of an in vitro evaluation, it was proven that the GO-PLGA membrane supported the proliferation of bone MSCs and their osteogenic differentiation. The local application of the GO-PLGA membrane to the space between the bone and the tendon in a rabbit model improved the healing enthesis, increased new cartilage and bone generation, and improved the CLG arrangement and biomechanical properties compared to the use of a PLGA membrane [91].

**Figure 4:**
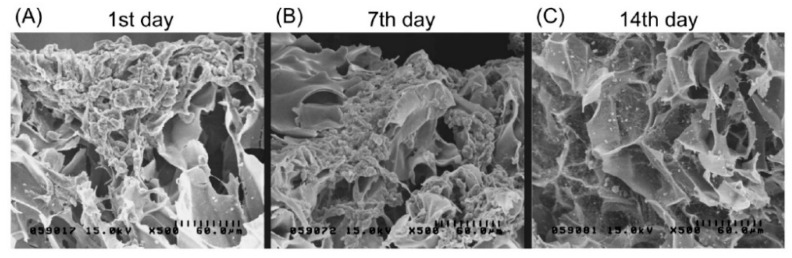
SEM images presenting chondrocytes (bearing magnetic nanoparticles) grown on the biphasic type II collagen–chitosan/polylactic-co-glycolic acid (CLG–CS/PLGA) scaffold at 1, 7, and 14 days after seeding. (**A**) No attachment of the cells on the scaffold after one day was observed, only their collection on the scaffold surface was indicated, (**B**) after seven days, cells started to adhere and proliferate deep into the scaffold, (**C**) after 14 days, the extracellular matrix was secreted with a higher accumulation on the scaffold surface. Cell adhesion and spreading on the pores of the scaffold is clearly visible. Magnification 500×. Adapted from [93], MDPI, 2017.

CLG is commonly used for cartilage repair, but chondrogenesis is disfavored by its low stiffness and rapid degradation. An injectable hydrogel was prepared using biocompatible CDs and CLG, which were crosslinked by genipin (CLG-genipin-CDs, CGC) with higher stiffness. Using photodynamic therapy (PDT), a moderate amount of reactive oxygen species (ROS) was generated, which supported chondrogenic differentiation of bone marrow-derived MSCs and subsequently improved cartilage regeneration. The degradation rate of CGC was 39% lower and the compression modulus was 21-fold higher compared to the pure CLG hydrogel. The CGC hydrogel in combination with PDT enhanced the bone MSCs proliferation by 50.3%, and the cartilage regeneration was less than eight weeks [92]. A type II CLG–CS/PLGA scaffold was used for the cultivation of rabbit chondrocytes labelled by magnetic NPs to prepare cultures with visible cells to study their growth, differentiation, and regeneration. The SEM image showed no cell attachment on the scaffold after one day; the cells were only collected on the scaffold surface. After seven days, the cells began to adhere and proliferate deep into the surface of the scaffold. After 14 days, a secretion of extracellular matrix was visible together with the accumulation on the scaffold surface (see Figure 4). Magnetic NPs did not affect the chondrocyte phenotype or protein and gene expression. Increasing gene expression of aggrecan and type II CLG indicating chondrogenesis was observed. The differentiation was associated with osteogenesis [93].

Thermosensitive CS-based composites chemically modified with CLG and containing bioactive glass NPs were used for the preparation of injectable nanohybrids for regenerative medicine. The thermosensitive response of the hydrogel was approximately 37 °C, which corresponds to the human body temperature. The CS hydrogels were characterized by 3D-porous structures; the presence of CLG increased the average pore size and together with the presence of bioactive glass improved the mechanical properties. The addition of 2 wt% of bioactive glass NPs led to an approximately 39% increase in stiffness compared to pure CS, while the addition of 30 wt% of CLG increased the stiffness by 95%. No toxic effect of the composites on the human osteosarcoma cell culture and kidney cells line of human embryo (HEM293T) was found using MIT (170 μL, 5 mg/mL; Sigma-Aldrich, St. Louis, MO, USA) and Live/Dead^®^ assays (Life Technologies of Brazil Ltda, São Paulo, Brazil) [94].

For cartilage repair, hydrogels having enhanced biocompatible, biotribological, and biomechanical properties also seem to be perspective materials. Physically cross-linked PVA-nHA/(2-hydroxypropyltrimethyl ammonium chloride CS) hydrogels with a double network were developed via a freezing/thawing technique and an immersing process. The resulting hydrogel with an optimized HA content exhibited outstanding mechanical properties such as fracture tensile stress (2.70 ± 0.24 MPa), toughness (14.09 ± 2.06 MJ/m^3^), and compressive modulus (0.88 ± 0.09 MPa) accompanied with notable anti-fatigue property, exceptional self-recovery, and the ability of energy dissipation, which was caused by these cross-linked structures. The content of nHA positively influenced the low value of the friction coefficient and the excellent cytocompatibility [95]. A bi-polymeric PVA/polyvinylpyrrolidone hydrogel composite with incorporated stick-like TiO_2_ nanostructures was designed. The resulting hydrogel composites had an improved surface topography, and more flatted cell morphologies and enhanced osteoblast attachment were observed. The stick-like TiO_2_ NCPs and crystalline bone promoted the bioactivity via lamellipodia and filopodia extension of osteoblast cells because of their excellent intercellular connection and regulated cell responses. An antibacterial activity against *Staphylococcus aureus* and *Escherichia coli* bacterial strains was also found [96].

Biocomposites of glycol (GLY)–CS matrices containing nHA with the average size of 74 ± 15 nm were fabricated by an eco-friendly chemical colloidal process in aqueous media, solvent casting, and evaporation at room temperature. It was found that the GLY–CS ligand had a major role in the nucleation, growth, and colloidal stabilization of nHA. nHA particles were homogenously dispersed in the GLY–CS matrix. An adequate cell viability response and non-cytotoxic behavior toward osteoblastic-like and embryonic cell lines (HEK293T) were proven. Based on the osteogenic differentiation tests, it is obvious that the nHA/GLY–CS composites are osteoinductive for human bone MSCs and can be tested for bone, cartilage, and periodontal regeneration [97]. Novel in situ forming composite hydrogels based on CS and GLT biopolymers associated with bioactive glass NPs were synthesized and characterized by the zeta potentials at 37 °C ranging from +3.1 ± 1.4 mV to +6.9 ± 3.2 mV. The cationic nature of these biocomposites was confirmed with the ability of interaction with anionic compounds contained in the native extracellular matrix. FTIR spectra showed that the hydrogels form a network mainly through molecular interactions. The elastic modulus (G) increased from 5.4 Pa for pure CS hydrogels to 12.4 Pa for the composites with higher GLT and bioactive glass contents. All formulations were injectable and cytocompatible, as confirmed by the live cell viability responses of the human osteosarcoma cell line [98]. ChS loaded zein NPs (~150 nm) were incorporated in a hydrogel based on a biphasic semi-interpenetrating polymer networks formed by blending ALG with PVA and calcium crosslinking. The final hydrogel system was used for functional articular hyaline cartilage restoration. The hydrogel was characterized by a porous microstructure with a 39.9 ± 5.8 μm pore diameter and 57.7 ± 5.9% porosity, swellability of 92%, and an elastic modulus higher than > 350 Pa. Compatibility with primary chondrocytes, interaction of chondrocytes with the matrix, and cell–cell clustering were studied; proliferation was determined; and positive influence of ChS on chondrocytes was proven [99]. Yang et al. [100] used the nonprotein compound kartogenin (KGN), which is able to promote the differentiation of bone marrow-derived MSCs into chondrocytes. KGN was anchored onto the surface of modified superparamagnetic iron-oxide (SPIO) and incorporated with cellulose nanocrystal/dextran hydrogels, which served as a carrier for SPIO–KGN as well as a matrix for the repair of cartilage. It was found that KGN is released stable in the long run, intakes endogenous host cells, and promotes bone MSCs to differentiate into chondrocytes. Thus, it is suitable for a cartilage regeneration. The regenerated cartilage tissue was similar to a natural hyaline cartilage [100].

All of the above-mentioned NCPs and their properties are summarized in Table 1.

## 4. Nanomaterials for Bone Healing and Regeneration

Nanotechnology has shown a revolution in tissue engineering and bone healing. Combinations of the benefits of nanomaterial design and synthesis, along with progress in genomics, proteomics, and tissue engineering, have brought new possibilities for orthopedic traumatology and bone healing. Dozens of applications were studied using nanometric entities, structures, and devices. Scaffold synthesis, delivery systems, controlled modification of surface topography and composition, and biomicroelectromechanical systems have been demonstrated in many biomedical studies [101]. Scaffolds based on nanomaterials and NCPs with their nanoscaled structures and topologies mimicking the physiological characteristics of natural bone tissue are very promising for promoting the formation of new bone tissues. These can reach excellent biocompatible and osteogenesis characteristics and can play a vital role in bone regeneration [102,103]. Generally, bone possesses a capacity to fix itself. However, in the case of a larger defect, external solutions such as autografts must be applied, which can have some negative effects such as donor-site morbidity. Porous biodegradable scaffolds provide an external support for cell growth, and finally, they degrade when the defect is repaired. The main requirements of the properties of such scaffolds involve biocompatibility, interconnected porosity, suitable mechanical properties, and biodegradability. Additive manufacturing methods are a very promising solution allowing tailored 3D printed composite-based scaffolds [104].

Among the materials that are used or being developed as potential implants, HA should be mentioned first. It can be combined with other materials to improve its mechanical and biological properties. Furthermore, various carbon-based nanomaterials and ceramic materials based on silicon and aluminosilicates are widely used. Inorganic materials based on magnesium, iron, and titanium are also being developed. Of course, combinations of all of these above-mentioned components can also be found in order to create an ideal implant with optimal mechanical and biological properties.

### 4.1. Nano-Hydroxyapatite

Nano-hydroxyapatite (nHA) is a very promising bioactive material due to its biocompatibility. One of its main advantages is its similarity to the inorganic bone structure with outstanding physical, chemical, mechanical, and biological properties. The functional and structural properties of nHA can be controlled during NP synthesis. It is used in various applications such as bone tissue engineering, implantology, surgery, periodontology, esthetics, and prevention, for example, as a coating material for titanium implants in dentistry also showing antibacterial activity, as a grafting material, or as material with remineralizing potential [105,106]. It can be isolated from bio-waste materials, for example, Fariborz et al. [107] used the ball milling process after annealing waste pigeon bones at 850 °C followed by cold-pressing of the NPs and resintering at 850, 950, 1050, and 1150 °C for nHA preparation. The average particle size of the prepared nHA was in the range of 50–250 nm; the Ca/P ratio (sintering at 1050 °C) was 1.7; hardness and compressive strength of sintered nHA were increased to 47.57 MPa and 3.7 GPa, respectively. A significant improvement in the activity and proliferation of osteoblast cells was proven compared to synthetic nHA [107].

**Figure 5:**
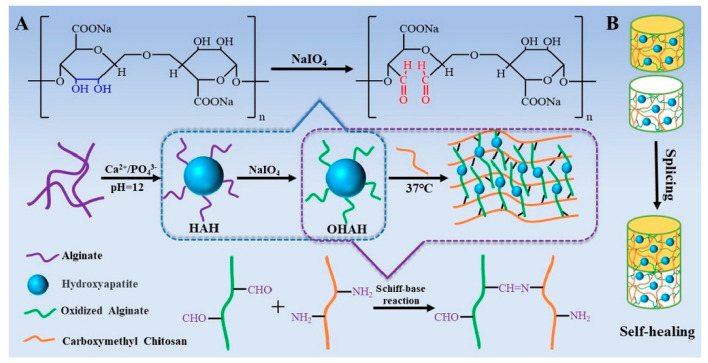
Schematic illustration of the preparation of the hydrogel based on alginate (ALG), hydroxyapatite (HA), and carboxymethyl chitosan (CM)–CS. ALG was used for the preparation of HAH hybrids, then ALG was oxidized to form oxidized OHAH hybrid. In the next step, the Schiff base reaction between OHAH and (CM)–CS was launched. The self-healing property of the hydrogel was verified using two parts of the prepared hydrogels, one of the parts was mixed with methyl orange solution. Both parts were then spliced and characterized. Scheme describes: (**A**) the preparation of an injectable hydrogel using Schiff base reaction via chemical reactions as well as schematically, (**B**) the self-healing property of the hydrogels. HAH is hydroxyapatite alginate (HA/ALG) hybrids, OHAH is oxidized alginate hydroxyapatite hybrid. Adapted from [108], Elsevier, 2017.

The injectable hydrogels based on oxidized ALG hybrid HA NPs and carboxymethyl (CM)–CS were prepared. The formation of the hydrogels based on the dynamic imine bonding via the Schiff base reaction was confirmed using rheological measurements, and their self-healing property was validated by the splicing experiments and rheological experiments. The structure of hydrogels was porous with HA NPs distributed on the surface of pore wall and they were cytocompatible. Figure 5 presents an illustration of the preparation of the injectable hydrogel via the Schiff base reaction as well as the self-healing property of the hydrogels [108].

Composite nanofiber membranes compatible with BMSCs were prepared using poly(d-lactic acid)-grafted HA and enantiomeric PLA. The tensile strength and Young’s modulus of the composite nanofiber membrane was increased by 30.16% and 34.56%, respectively. The proliferation and adhesion of bone MSCs cultured on the nanofiber membranes together with increased type I CLG expression and the improved formation of bone-like nodules were confirmed [109]. Mesoporous silica Santa Barbara Amorphous-15 (SBA15) and nHA were incorporated into the PLLA scaffold produced using selective laser sintering. Silicon and calcium were released due to the SBA15 degradation and nHA stimulated cell response. Moreover, the hydrated silica gel layer could adsorb calcium ions released from nHA. A good biomineralization capacity as well as a good cell response via the evaluation of the cell attachment and the alkaline phosphatase (ALP) activity expression was observed. SBA15 and nHA increased the scaffold hydrophilicity (the measured water contact angle raised from 107.4° to 57.8°), but the acidic hydrolysate of PLLA was also neutralized [110]. A photocrosslinkable NCP ink consists of tri-block poly(lactide-co-propylene glycol-co-lactide) dimethacrylate P_m_L_n_DMA (m and n denoted the unit length of propylene glycol and lactide) and hydroxyethyl methacrylate (HEMA)-functionalized HA NPs (nHAMA). The nHAMA interacted with P_m_L_n_DMA upon light exposure and an inorganic–organic co-crosslinked NCP network was formed. Mechanical properties of the prepared NCPs were highly enhanced compared to the polymer (e.g., compressive modulus increased by nearly 10-fold from approx. 40 to approx. 400 MPa). It was found that they produced low exothermic heat generation (<37 °C) during photocrosslinking, thus, they could easily encapsulate and ensure the long-term release of heat-labile bone morphogenetic protein (BMP)-2. The advantages were also tunable rheological properties, wettability, degradation, and 3D printability [111].

A mesoporous HA surface modified by poly(γ-benzyl-l-glutamate) (PBLG) with different amounts (from 11 to 50 wt%) was prepared using the in situ ring opening polymerization of γ-benzyl-l-glutamate *N*-carboxy anhydride. Then, PBLG-g-mesoporous HA/PLGA composite films and porous scaffolds were prepared to demonstrate the biological performance of the composites. In conclusion, it was found that the in vivo rabbit radius defect repair showed rapid mineralization and new bone formation by using the composites with 22 and 33 wt% PBLG [112].

The simple fibers of acrylate epoxidized soybean oil/PEGDA/nHA-based NCPs showed a significant improvement in their mechanical properties when extruded with smaller needles before curing by UV radiation. It was confirmed by SEM that the nHA were well dispersed in the polymer matrices. The ultimate tensile strength and moduli increased with the decrease of the extrusion needle diameters, which correlated with higher matrix crystallinity and fewer defects. For example, the filaments extruded via the needle diameter of 0.84 mm showed the tensile stress and modulus of 26.3 ± 2.8 MPa and 885 ± 100 MPa, respectively; filaments extruded via needles with the diameter of 0.21 mm showed the ultimate tensile stress and modulus of 48.9 ± 4.0 MPa and 1696 ± 172 MPa, respectively [113]. The nHA/CS/poly(methyl vinyl ether-alt-maleic anhydride) (nHA/CS/P(MVE-alt-MA)) composite was fabricated via electrostatic interaction. nHA was uniformly distributed in the polymer matrices CS/P(MVE-alt-MA), and the NCP mechanical properties were better than those of single components; the maximum compressive strength was up to 8.48 MPa. This NCP also showed outstanding biocompatibility using Sprague–Dawley (SD) rat bone MSC culture [114]. Electroactive and bioactive 3D porous NCP scaffolds were synthesized by a freeze-drying method using 1,4-dioxane as a solvent. The copolymer (PAP_(n)_) was prepared using the condensation polymerization of hydroxyl-capped PLA and carboxyl-capped aniline pentamer (AP). A HA grafting l-lactic acid oligomer (op-HA) was used as a bioactive component and showed a better interface compatibility with PAP_(n)_ and PLGA. A good biocompatibility was shown for these implants with higher osteogenetic activity by promoting cell ingrowth and CLG fibers forming. The composite scaffold containing 1 wt% PAP_(n)_ demonstrated more suitable properties (e.g., a distinct bone callus, bridging growth, vague borderlines between newly formed bone at the two defect ends, and increased bone density) [115].

Nanocrystalline nHA-poly(thioketal urethane) (PTKUR) cements were used for femoral defect treatment in New Zealand White rabbits to study ossification at 4, 12, and 18 months. Four samples of cements were tested: injectable, flowable cement, and three moldable putties containing varying ratios of calcium phosphate to sucrose granules. The formation of new bone and cement resorption by osteoclasts were confirmed near the periphery. Chondrocyte infiltration into the cements and ossification of the cartilaginous intermediate was proven via Stevenel’s Blue and Safranin O staining. nHA–PTKUR cements positively influenced combined intramembranous and endochondral ossification, leading to enhanced osseointegration of the cement [116]. Poly(butylene adipate-co-terephthalate) (PBAT) was mixed with different concentrations of nHA (1, 2, 3, 4, 5, and 6 wt%) solutions to produce scaffolds thorough electrospinning. A reduction in crystallinity was observed with the increasing nHA concentration. There was no cytotoxicity found in the tests of all scaffolds, and all the prepared PBAT/nHA scaffolds supported bone repair [117].

#### Metal Doped Hydroxyapatite

AuNP-loaded HA NCPs were fabricated to control the osteogenic differentiation of human MSCs via the synergistic effects of both AuNPs and HA. The HA–AuNPs exhibited a good cytocompatibility and were internalized into human MSCs. The increase in human MSC osteogenic differentiation was confirmed by the increased ALP production level, calcium mineralization deposition, and the typical osteogenic gene expression. The Au incorporation activated the Wnt/β-catenin signaling pathway. A synergistic effect on human MSC osteogenic differentiation was exerted using the HA–Au NPs [118]. Microspheres (COS–Ag–ALG-HA) with size ranging from 1.5 ± 0.5 to 4.0 ± 0.5 mm and involving chitooligosaccharide (COS) coated AgNPs with ALG and HA were designed and prepared. The prepared microspheres were rigid with mutual chemical interactions between individual parts. High antimicrobial activity was observed against *S. aureus* together with the biocompatibility with osteoblast-like cells [119]. Zinc-doped HA could be used as a graft biomaterial for bone regeneration, but the Zn effect on osteoconductivity has still been unknown due to the Ca, P, and Zn release and resorption in graft-implanted defects. Microspheres consisting of ALG and non-doped carbonated HA or ALG and nanocrystalline 3.2 wt% zinc-doped HA (Zn–HA) were inserted in critical-sized calvarial defects in Wistar rats for one, three, and six months. Any significant difference in the new bone quantity was not determined between these two materials, and they both released high Ca, P, and Zn quantities, which were distributed in the defective area. Zn was strongly adsorbed by the HA surface. Phosphorus was resorbed faster than Ca. Zn and Ca showed equivalent release profiles, which confirms their stoichiometric dissolution and non-preferential Zn resorption. The high nanometric Ca and Zn accumulation in the defect influenced osteoconduction, inhibiting and impairing bone repair [120]. The porous scaffolds of the ZrO_2_/HA composite were formed by the digital light processing (DLP) technology with a positive effect on cell proliferation and differentiation. The scaffold containing 10 wt% HA had the best compressive capacity. After the scaffold was immersed in the simulated body fluid, its compressive strength decreased within the first 14 days and then increased probably due to the degradation of calcium phosphate components and the deposition of apatite. On day 28, the compressive strength reached approx. 20 MPa and was close to that of the scaffolds made of ZrO_2_ (25 MPa) [121].

A protein corona formation can help to understand the mechanisms of immune-modulated bone wound healing. An in vivo dynamic model for the protein corona of magnetic HA scaffolds was designed to study the correlation between the inflammatory reaction and bone wound healing together with the underlying mechanism controlling this process. The levels of some proteins related to the immune response and inflammation, bone and wound healing, extracellular matrix, cell behavior, and signaling were increased in the protein corona of the MNP-infiltrated scaffolds in a time-dependent manner. The immune response and inflammation proteins adsorbed on the magnetic HA scaffolds correlated well with the bone wound healing proteins. The presence of MNPs suppressed the chronic inflammatory responses, but highly promoted the acute inflammatory responses. The activation of acute inflammatory reactions induces the recruitment of immune cells and remodeling of the extracellular matrix, which leads to accelerated bone healing [122]. A NCP scaffold was synthesized using bacterial cellulose (BC) with magnetite (Fe_3_O_4_) and HA NPs using ultrasound. The resulting scaffold (BC–Fe_3_O_4_–HA) had homogenous dispersion of the NPs in the BC matrix with a Ca/P ratio of 1.63 and 1.56 for the surface and cross section, respectively. The BC crystallinity index was lowered in the composite (from 82.5% to 62%). A decrease in saturation magnetization from 15.84 to 3.94 µ/g at ± 10 kOe was found after the deposition of HA with superparamagnetic characteristics together with significant lowering in swelling ability after the incorporation of the NPs and high porosity degree (around 80%). The scaffold was non-toxic to mouse fibroblast L929 cells and biocompatible for osteoblast (MC3T3-E1 cell line) attachment and proliferation [123].

A comprehensive review of the current issues of preparation and properties of magnetic HA and the application of these NCP materials in biomedicine as implants for bone regeneration with antimicrobial activity, controlled drug/gene delivery, and magnetic hyperthermia treatment was recently published by Mushtaq et al. [124]. Additionally, Scialla et al. [125] obtained positive results of the use of a NCP composed of magnetic iron oxide grafted with dextran in combination with nHA in bone tissue engineering [125].

All of the above-mentioned NCPs and their properties are summarized in Table 2.

### 4.2. Carbon-Based Nanomaterials

A scaffold based on carbon nanomaterials such as GO, CNTs, CDs, and their derivatives, has become one of the key materials, which, depending on their functionalization, has remarkable abilities to influence bone regeneration, effective cell proliferation, and osteogenic differentiation. Thus, CNTs and GR-based nanomaterials have often been tested as nanoreinforcements in bone tissue engineering due to their unique mechanical, electrical, and biological properties, allowing them to be secondary-phase reinforcements. In addition, NCPs containing CNTs and GR demonstrated better osteoblast cell adhesion, leading to the promotion of bone tissue formation in vivo; thus, they are expected to bring groundbreaking technologies to regenerative medicine and bone tissue engineering [33,126]. Furthermore, they provide antimicrobial properties and can reinforce the mechanical properties [104]. On the other hand, the problem is that the cytocompatibility of CNTs and GR is still a controversial topic [33,126].

#### 4.2.1. Carbon Nanotubes

A PLLA/CNT scaffold is a promising candidate as a bone implant. PLLA is a promising implant material due to its biocompatibility and degradability; however, the insufficient mechanical strength is not suitable for bone repair application. The crystallinity of PLLA scaffolds containing CNTs increased significantly because CNTs promote orderly stacking of PLLA chains. Moreover, CNTs acted as a bridge across the cracks. The compressive strength, Vickers hardness, and tensile strength of the scaffold were enhanced by 22.7%, 58.8%, and 17.6%, respectively [127]. Scaffolds based on electrospun PLLA matrix covered with hybrid composites of CNT/graphene nanoribbons (GNRs) and nHA were prepared and studied using various methods (SEM, EDS, and AFM). The GNRs showed a toxicity and cytotoxicity at the concentrations of 60 and 120 µg/mL, and neither toxicity nor cytotoxicity was determined at the concentration of 30 µg/mL using the *Allium cepa* assay. The hemolysis test determined that the scaffolds with the concentration of 0.3 mg/cm^2^ were not toxic, and corroborating data from the biochemical markers glutamic pyruvic transaminase, glutamic oxaloacetic transaminase, and urea showed no cytotoxicity, genotoxicity, or mutagenicity [128]. Ordered CNT–HA scaffolds with improved mechanical properties and accelerated cell growth in vitro or in vivo were prepared using agarose gel electrophoresis to imitate a pattern of CLG and HA hydrogel scaffolds (AG-CLG-o-CNT). The enhanced proliferation and differentiation of bone MSC lines was proven, and the bone defects were repaired after 28 and 56 days in vivo [129]. Osteogenic peptides have the osteogenic ability of artificial bone materials. CNTs with carboxyl and amino groups were used as a nanoreinforcement for synthetic scaffold materials, in which they were covalently attached to the RGD/BMP-2 osteogenic peptide. MC3T3-E1 cells were subsequently cultured on these scaffolds. The peptide bound via amino groups could promote cell functions more efficiently than that bound through carboxyl groups, probably due to the positive charges of the amino groups on the CNT surfaces, leading to changes in the peptide conformation, protein adsorption, and targeting osteogenic effects [130]. An injectable CNT and two-dimensional (2D) black phosphorus (BP) gel with enhanced mechanical strength, electrical conductivity, and continuous phosphate ion release was prepared. Biodegradable oligo(poly(ethylene glycol)fumarate) polymer was used as a hydrogel cross-linking matrix together with the cross-linkable CNT–poly(ethylene glycol)acrylate (CNT–PEGA) to improve the mechanical properties and electric conductivity. The BP–CNTPEGA gel enhanced the adhesion, proliferation, and osteogenic differentiation of MC3T3 preosteoblast cells. The osteogenesis of preosteoblast cells was improved with electric stimulation [131].

Du et al. [132] found that multi-walled carbon nanotubes (MWCNTs) could be more effective for enhanced bone formation than nHA. They studied the osteogenic ability of MWCNTs and nHA for the in vitro culture of human adipose-derived MSCs. No significant difference between the MWCNTs and the nHA was found in the cell adhesion amount; however, the cell attachment strength and proliferation of the MWCNTs were better. The MWCNTs also showed better induction of the HASC osteogenic differentiation than the nHA, and unlike the nHA, they could induce ectopic bone formation in vivo. It is assumed that MWCNTs concentrate more proteins such as specific bone-inducing proteins, which are secreted from M2 macrophages, and therefore stimulate inducible cells in tissues to form inductive bone better than nHA [132]. 3D conductive scaffolds made from PCL and MWCNTs were produced using extrusion-based additive manufacturing to treat large calvary bone defects in rats. Based on histology results, it was found that a combination of PCL/MWCNTs scaffolds and exogenous electrical stimulation induced thicker and increased bone tissue formation within the bone defect supported by promoted angiogenesis and mineralization with the concentration of MWCNTs of 3 wt% and electrical stimulation. The tartrate-resistant acid phosphatase positive cell formation was promoted. While the osteoclastogenesis was inhibited using MWCNTs, the use of ES promoted it [133]. Similarly, a 3D printed porous scaffold with aligned MWCNTs and nHA was prepared by Huang et al. [134]. MWCNTs with similar dimensions as CLG fibers coupled with nHA were mixed with a PCL matrix. It was confirmed that MWCNTS were aligned in the PCL matrix, and the scaffold was similar to the native bone nanostructure [134]. PCL scaffolds with double fillers, MWCNTs and eggshell, with improved mechanical and osteogenic properties were prepared. It was found that eggshell improved the PCL/eggshell/MWCNT scaffold hydrophilicity and biocompatibility, whereas MWCNTs enhanced their compression and tensile strength [135].

A tough polyion complex (PIC) hydrogel containing MWCNTs was synthesized to form a PIC/MWCNT biohybrid hydrogel, which was used for the fabrication of 3D scaffolds by extrusion-based 3D printing. The resulting scaffolds had a good biocompatibility with rat bone marrow-derived MSCs and enhanced their osteogenic differentiation. A higher degree of osteogenic differentiation was obtained by using PIC/MWCNT scaffolds than PIC scaffolds. In addition, the PIC/MWCNT scaffolds significantly promoted the regeneration of calvarial defect healing [136].

Nanocrystalline cellulose is a widely available natural material on Earth. It is isolated from lignocellulosic plants or from agricultural waste using the acid hydrolysis method. It can be characterized by outstanding physicochemical properties, low toxicity, and ecotoxicological risks toward living cells. Due to these facts, it has often been used in designing materials of bone scaffolds [137]. A novel NCP scaffold based on nitrogen-doped MWCNTs, cellulose, and nHA was designed by Xing et al. [138]. The mechanical properties of the hybrid scaffold containing 1 wt% N-MWCNTs were significantly improved, and its surface morphology was rough and porous. In vitro cellular attachment, proliferation, viability, and mineralization of bone MSCs was also confirmed. The presence of N-MWCNTs in the scaffold induced the preferential differentiation of bone MSCs to osteogenic lineage, which was accompanied by increased ALP activity and the expression of key osteogenic genes. Not only was the interface bonding with the bone tissue accelerated, but new bone formation and regeneration were also confirmed [138].

#### 4.2.2. Graphene-Based Materials

GR and GO are able to support cell growth and proliferation, cell attachment, and cytoskeleton development and to activate osteogenesis and bone development. They also have positive effects on a polymer matrix causing more ordered morphologies, greater surface area, and higher total porosity, which are favorable scaffold properties facilitating cell attachment and migration [139].

**Figure 6:**
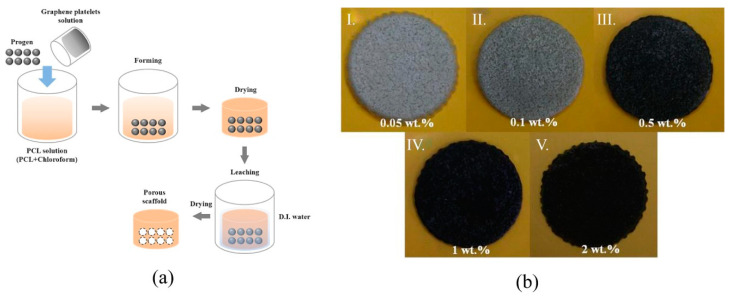
Illustrations of polycaprolactone/graphene platelet scaffolds preparation. Graphene (GR) platelets were prepared using graphite via intercalation at 700 °C for 60 s. The formed GR platelets were dispersed in trichloromethane and mixed together with NaCl (progen) with a polycaprolactone solution (PCL), stirred for 2 h, and poured into a form. After drying, the system was immersed in deionized water (D.I.) to remove NaCl and consequently dried to form a porous scaffold. Various GR amounts were used for the scaffold preparation. (**a**) Scheme of PCL/GR scaffold preparation and (**b**) scaffold samples with various contents of GR platelets I. 0.05 wt%, II. 0.1 wt%, III. 0.5 wt%, IV. 1.0 wt%, and V. 2.0 wt%. Adapted from [141], Elsevier, 2020.

HA/hydrophilic GR (hGR) composites with a higher stability were prepared without extra ion introduction using the self-assembling method. The crosslinked structure was formed due to the internal interaction between HA and hGR, and the composite roughness and hydrophilic ability could be tailored using an increased hGR content. The composite HA/5%hGR demonstrated a higher cell proliferation rate (264.81%) and supported the spreading and growth of MC3T3-E1 cells compared to the pure HA [140]. Polymeric hybrid NCPs containing carrageenan/acrylic-acid/GR/HA and mimicking the structural and chemical composition of natural bone were synthesized using free-radical polymerization and intended for fractured bone regeneration. Structural properties, surface morphology, hydrophilicity, biodegradability, and swelling of the NCPs together with the cell viability, cell culture, and proliferation against mouse preosteoblast (MC3T3-E1) cell lines were tested. Optimum porosity of 49.75% and pore size of 0.41 × 10^3^ µm^2^, mechanical properties such as compression strength of 8.87 MPa and elastic modulus of 442.63 MPa, swelling of 70.20% at 27 °C, 77.21% at 37 °C, and biodegradation of23.8% were confirmed [103]. GR platelets as fillers, NaCl as a porogenic material and PCL as a matrix were used to produce porous scaffolds using the solvent-casting/particulate-leaching method. The preparation process and products are shown in Figure 6 [141]. The compressive strength, porosity, contact angle, weight loss, and variations in pH values in degradation tests as well as the biocompatibility by seeding osteoblast-like (MG-63) cells in vitro were studied, and it was confirmed that the mechanical properties, cell attachment, and proliferation were improved with a higher ratio of GR [141].

GR nanosheets and polyether ether ketone (PEEK) were used for the preparation of multifunctional NCPs with 12 orders of magnitude increase in electrical conductivity due to the formation of an electrical percolation network and π–π* bonds between GR and PEEK. This supported electrophoretic deposition of a bioactive/antibacterial coating consisting of stearyltrimethylammonium chloride-modified HA. The resulting coated implant demonstrated significant boosting of bone MSC proliferation in vitro with the photothermal conversion effect of the GR nanofillers. It is usable for photothermal applications such as increasing bacterial eradication, tumor cell inhibition, or bone tissue regeneration in vivo [142].

PEEK NCPs with various GO loading were prepared by injection molding. The GO loading influenced selected mechanical properties, and the greatest elongation at break (86.32% higher than that of pure PEEK) was in the case of 0.5% GO probably due to the well dispersed GO forming π–π* bonds with PEEK. The increasing GO content (>0.5%) induced GO agglomeration and, consequently, the deterioration of some mechanical properties. The addition of GO into PEEK supported the adhesion and spreading of bone MSCs [143]. Huang et al. [144] described a NCP composed of PEEK, in which GO and HA were incorporated. After laser treatment, the composite had surface macropores with diameters from 200 μm to 600 μm, which improved cell adhesion and proliferation of constant cells and thus overall biocompatibility and utilization [144]. Lopes et al. [145] prepared HA–GO NCPs with the addition of 0.5 wt%, 1.0 wt%, and 1.5 wt% of GO. HA NPs were adhered to the surface of the GO sheets, and the affinity between HA and GO increased from 0.5 wt% to 1.5 wt% GO in the HA–GO NCPs. The bioactivity properties of HA–GO NCP and indirect cytotoxicity connected with a decrease in the human dental pulp stem cells viability and proliferation occurred when GO concentration increased to 1.5 wt%. Thus, the 0.5 wt% HA–GO NCP was a promising biomaterial for bone tissue regeneration compared to the pure HA [145]. The GO/CS/nHA scaffold was prepared via the effective regulation of CS functionalized with a GO network matrix and demonstrated enhanced properties such as 3D porous bone-like hierarchical structure, proper mechanical property, and biodegradation as well as suitable water uptake and retention ratio. The biomimetic mineralization and cell culture experiments demonstrated that the hybrid scaffold possessed superior bioactivity and cell proliferation ability in vitro. In addition, the rat calvarial defect repair models and tissue pathological characterization further proved that the hybrid scaffold had excellent biocompatibility and the capability to induce bone regeneration in situ. The prepared scaffold might be an excellent candidate for endogenous bone repair [146]. The nanohybrids of 2D rod-like nHA loaded on a low-concentration GO sheet (GO–nHA) were inserted into spermine-based high-strength thermoplastic polyurethane-urea (PUU) matrices using an in situ technique to fabricate porous scaffolds. The scaffolds with the content of 1 wt% GO–nHA showed improved physico-mechanical properties. Cytotoxicity tests using osteoblast cells such as the MG-63 cell line confirmed cell viability above 95% and improved proliferation over a period of two weeks of culture. Type I CLG expression was positive, and perfect maturation and biomineralization of osteoblasts was indicated by osteocalcin (OCN) presence [147]. GO and isocyanate were used to prepare a GO shape-memory polyurethane composite with improved mechanical and shape-memory effects. The modulus of approx. 339 MPa and the shape recovery ratio of 98% were obtained. After being implanted in a defective bone via a minimally invasive treatment, the composite ensured a generated force during the recovery process and seemed to provide a new possibility for a practical application of shape-memory polymers and composites in the field of bone repair [148]. nHAp/CNTs with GO and termed GNRs composites had good bioactivity and osseointegration properties for bone regeneration. Three different contents of GNR (1, 2, and 3 wt%) in nHAp/GNRs were used. The assessment was made in vivo using 36 Wistar rats with osteoporosis induced by oophorectomy in female rats prior to implantation. The evaluation was made after days 21 and 45, when histological, biochemical, and radiographic analyses (DIGORA method) were done and evaluated through ANOVA, the Tukey’s test, and the Kolmogorov–Smirnov test with statistical significance at *p* < 0.05. The osteoconductive activity of nHAp and GNRs was observed in dependence on GNR concentration in the following order: 3 > 2 > 1 wt% [149].

It is known that the strontium (Sr)-substituted HA scaffold cannot properly fit the required mechanical properties. GO-reinforced SrHA NPs were prepared using a hydrothermal method. GO easily self-assembles into a layered structure in the dispersion, which helps to regulate the SrHA deposition on the GO surface. The SrHA/GO NPs were then used for incorporation into CS and quaternized chitosan (qCS) mixed solutions to prepare the scaffold by a freeze-drying method. The compressive modulus of the CS/qCS/SrHA/GO scaffold achieved 438.5 kPa, being 4-fold higher than that of the CS/qCS scaffold. In addition, in vitro mineralization levels and ALP activity were increased [150]. Sr–GO NCPs allowing for the long-term release of Sr ions were fabricated and subsequently used to reinforce CLG scaffolds. The resulting Sr–GO–CLG scaffolds showed high water retention rates and excellent mechanical properties. They displayed a strong effect on adipose-derived stem cells, which was obvious from cell adhesion and osteogenic differentiation, and promoted the secretion of angiogenic factors to stimulate the in vitro tube formation of endothelial cells. The angiogenic vascular endothelial growth factor (VEGF) and osteogenic BMP-2 protein secretion were increased due to synergistic effects of GO and Sr. If transplanted into rat critical-size calvarial bone defects, the best bone regeneration and angiogenesis were observed at 12 weeks. In addition, results showed that the Sr–GO–CLG group achieved complete defect bridging with the newly formed bone tissue, and the residual Sr–GO NPs were phagocytosed and degraded by multinucleated giant cells [151].

Aerogels based on natural polymers have high porosity and great biocompatibility; however, their mechanical properties are extremely poor when using them as an artificial graft for bone repair. A highly porous and hydrophilic aerogel was formed by GO and type I CLG using the sol-gel process (GO content: 0, 0.05, 0.1, and 0.2% *w*/*v*). The compressive modulus increased with the higher GO content. The 0.1% GO–CLG showed better biomineralization rate and cell compatibility in vitro, while a better bone repair effect compared to that of CLG aerogel was observed in rat cranial defect models in the in vivo study [152]. A bioactive PLGA-α-tricalcium phosphate (α-TCP) composite scaffold containing GOs and BMP-2 peptide (PTG/P) was produced by a cryogenic 3D printing method to repair a critical-sized bone defect. The scaffolds were comparable to human cancellous bone with its mechanical properties and hierarchical porosity. GO enhanced the scaffold wettability and mechanical strength; the peptides ensured biological activity. The rat bone marrow-derived MSC ingrowth into the PTG/P scaffold and enhanced osteogenic differentiation were promoted [153]. Silk fibroin (SF) is a natural protein without any physiological activity, which has good biocompatibility, is easily processed, and causes minimal inflammatory reactions in the body. SF electrospun scaffolds containing GO functionalized with BMP-2 polypeptide were prepared via electrostatic interactions. The resulting scaffold showed a better biocompatibility, promoting cell adhesion and proliferation and enhancing in vitro the osteogenic differentiation of bone marrow stromal cells using either an osteogenic or non-osteogenic medium. In vivo bone formation in critical-sized calvarial bone defects was also proven [154]. MicroRNAs (miRNAs) are important for regulating osteogenic differentiation and bone formation. A polyethylenimine (PEI)-functionalized GO complex was loaded with the miR-214 inhibitor into SF/HA scaffolds. SF/HA/GO scaffolds showed high mechanical strength, and cell adhesion and growth were promoted. The SF/HA/GO–PEI scaffolds loaded with miR-214 inhibitor (SF/HA/GPM) enhanced osteogenic differentiation by inhibiting the expression of miR-214 and at same time by increasing the expression of activating transcription factor 4 (ATF4) and activating the Akt and ERK1/2 signaling pathways in mouse osteoblastic cells (MC3T3-E1) in vitro [155].

Polymer based ink materials are characterized by insufficient mechanical strength, low scaffold fidelity, and loss of osteogenesis induction. A human MSC-laden GO/ALG/GLT composite bioink was prepared to form 3D bone-mimicking scaffolds using a 3D bioprinting technique. The GO composite bioinks containing higher GO concentrations (0.5, 1, and 2 mg/mL) improved the bioprintability, scaffold fidelity, compressive modulus, cell proliferation, osteogenic differentiation, and extracellular matrix mineralization compared to the pure ALG/GLT system, while the bioink with GO concentration 1 mg/mL was the optimal filler [156]. A scaffold composed of mesoporous bioactive glasses and GO was investigated for local angiogenesis and bone healing. It had better cytocompatibility and higher osteogenesis differentiation ability with rat bone BMSCs compared to the pure mesoporous bioactive glass scaffold. It also supported vascular ingrowth and enhanced bone repair at the defect site in a rat cranial defect model. The newly formed bone was integrated not only on the periphery, but also in the scaffold center [157].

Fe_3_O_4_/GO NCPs were added into α-TCP/calcium sulfate (CaS) biphasic bone cement to prepare injectable magnetic bone cement (α-TCP/CaS/Fe_3_O_4_/GO, α-CFG) for the application in bone tumor minimally invasive surgery. The magnetothermal properties of the α-CFG bone cement could be well adjusted by changing the Fe_3_O_4_/GO NCP content and the magnetic field parameters. The most stable bone cement with excellent magnetothermal performance was in the case of 10 wt% content of Fe_3_O_4_/GO. The α-CFG bone cement enabled bone regeneration and demonstrated tumor treatment effects [158].

The hierarchical porous HA/rGO composite scaffolds were prepared using a soft template method with nanosurface morphology, suitable porosity and pore size, and good biomechanical strength. The loaded rGO promoted the adhesion, proliferation, and spontaneous osteogenic differentiation of bone MSCs. The scaffold is gradually degraded and newly formed bone replaces it [47]. A zinc-doped HA NCP decorated on rGO, named as G_3_H_ap_Z, showed its osteoconductive potential in biomineralization studies. The osteoinductive ability was tested on MSC differentiation to osteogenic lineage and expression of osteogenic markers including runt-related transcription factor 2 (RUNX-2), ALP, type I CLG, BMP-2, OCN, and osteopontin (OPN). Thus, the G_3_H_ap_Z NCPs were tested as orthopedic bone grafts to accelerate bone regeneration [159]. Shape memory polymers (SMPs) have a great potential for applications in the area of minimally invasive surgery. HA/rGO nanofillers were inserted into shape memory polyurethane (SMPU) to improve its mechanical properties. This NCP was further modified using arginyl-glycyl-aspartic acid (AGA) to improve cellular adhesion. It was observed that the mechanical properties of SMPU/HA/rGO/AGA NCP were significantly improved (e.g., a 200% increase in Young’s modulus and >300% enhancement in tensile strength compared to the unmodified SMPU). Rabbit bone MSCs were adhered on the NCP surface. The excellent shape memory behavior (e.g., shape fixity ratio 97.3% and shape recovery ratio 98.2%) was confirmed [160]. Methyl vanillate (MV) is known to promote the Wnt/β-catenin signaling pathway and induce osteoblast differentiation. GLT-rGO for MV delivery was prepared to realize the effective osteogenesis for bone repair. The biocompatibility of GLT-rGO was proven by easy cell absorption and distribution in the nucleus and cytoplasm. The MV has a positive influence on the BMSC osteogenesis in a concentration-dependent manner with a significant improvement at the concentration level of 1 µg/mL. It was confirmed by the ALP assay, Alizarin red S staining, immunofluorescence staining, and gene expression of related osteogenic markers that the MV/GLT-rGO weight ratio of 1:1000 obviously increased BMSC osteoinduction [161].

#### 4.2.3. Other Carbon-Based Nanomaterials

An electrospun nano-bio membrane from PVA, nano-demineralized bone matrix, and carbon NPs was prepared using an electrospinning machine. Tests with the MG63 osteoblast cell line showed 100% biocompatibility, and more viable cells present in the nano-biomembrane were observed as well as more apatite formation was confirmed using SEM. The content of carbon NPs (0.6 wt%) influenced mechanical properties, which were improved, achieving a tensile strength of 14.58 ± 0.13 MPa, elongation at break 13.87 ± 0.05%, and water absorption 36.84 ± 0.11% [162].

CDs are another carbon material carrying various reactive groups on the surface. They provide a unique surface to transport therapeutic genes and promote osteogenic differentiation. MiR-2861 has revealed osteogenic differentiation effects. Bu et al. [163] created ascorbic acid-PEI CDs loaded with miR-2861 by the microwave-assisted pyrolysis method. The resulting CDs had excellent fluorescence stability utilizable for fluorescence imaging in vitro and in vivo. The CDs were incorporated into bone marrow stromal cells (BMSCs) and distributed in the mitochondria, endoplasmic reticulum, lysosome, and nucleus. It was proven that the CDs efficiently transferred miR-2861 into bone MSCs in vitro, and the CDs with miR-2861 (CD@miR) had the strongest osteogenic effects because they acted synergistically. No cytotoxicity was found [163]. Zn^2+^-passivated CDs showed good osteogenic activity in vitro and in vivo. A 5 mm diameter calvarial bone defect model was created in rats and the Zn-CDs were used for the treatment of the critical bone defect. It was found that zinc gluconate (Zn-G) and the Zn-CDs promoted the survival of BMSCs when the Zn^2+^ ion concentration was 10^−4^ mol/L (Zn-G: 45.6 µg/mL) and 10^−5^ mol/L (Zn-CDs: 300 µg/mL) or below, respectively. Compared to the osteogenic capability, the ALP activity induced by the Zn-CDs was better than that by Zn-G. The area of calcified nodules was increased in the Zn-CD group. Thus, Zn-CDs reached the highest osteogenic effect at the concentration of 10^−5^ mol/L without affecting cell proliferation in long-term stimulation [164].

Lai et al. [165] found that carbon nanocages (CNCs) could improve the osteogenesis of BMP-2 (0.8 µg/mL) at the concentration of 20–80 µg/mL in a dose-dependent manner. A composite porous SF/CNC scaffold was investigated for the controlled delivery of BMP-2. An initial burst release of 23.9% and a release over the subsequent six days to about 47.7% were determined. The promotion of the osteogenic differentiation of bone MSCs was evaluated as dose-dependent, and it was found that the BMP-2/SF/CNC scaffold significantly improved the osteogenic differentiation of bone MSCs in vitro and promoted new bone formation in vivo [165].

**Figure 7:**
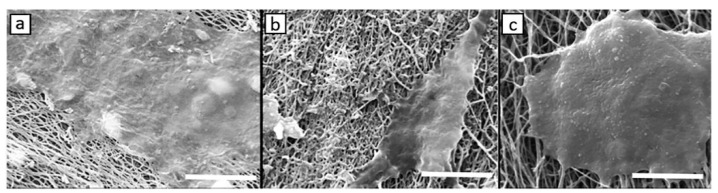
SEM images of MG-63 cell attachment and spreading on: (**a**) carbon nanofibrous (CNF) scaffolds without gold nanoparticles (CNF scaffold), (**b**) CNF scaffolds containing 2.5 wt% of gold nanoparticles (CNF/2.5% AuNPs), and (**c**) CNF scaffold with 5 wt% of sprayed AuNPs. Scale bar = 10 μm. Adapted from [166], Elsevier, 2020.

Carbon nanofiber (CNF)/AuNPs conductive scaffolds were prepared using blending electrospinning and electrospinning/electrospraying. The electrospun and electrosprayed nanofibers had decreased diameters: from 178.66 ± 38.40 nm to 157.94 ± 24.14 nm and 120.81 ± 13.77 nm, respectively. Electrical conductivity was increased by up to 29.2% and 81% by electrospraying and blending electrospinning, respectively. Neither significant toxicity nor influence on cell proliferation was observed. Cell attachment and spreading on prepared scaffolds are shown in Figure 7; their typical morphology and attachment have been found to be very promising for future studies [166].

Highly porous PEEK/HA bioNCP scaffolds reinforced with CNF and CNTs were prepared using salt porogen (size 200–500 µm) leaching methods. The NCP showed controlled pore size and distribution, enabling better cellular infiltration and the biointegration of NCPs within human tissue. It was proven that the NCPs were non-toxic with very good cell viability, and bone marrow cell growth was confirmed, while the presence of CNTs (0.5 and 1.0 wt%) and CF (0.5 wt%) increased cell attachment compared to the neat PEEK/HA composites, and the mechanical and biological properties were improved [167].

All of the above-mentioned NCPs and their properties are summarized in Table 3.

### 4.3. Silicates and Clays

A mesoporous silicate nanoparticle (MSN)-based electrospun PCL/GLT nanofibrous scaffold was prepared to ensure the delivery of alendronate (ALN) and silicate for modulating bone remodeling. ALN inhibits the bone-resorbing process through preventing guanosine triphosphate-related protein expression, and silicate induces the bone-forming process via improving vascularization and bone calcification. ALN was encapsulated into MSNs (ALN@MSNs) and then an acetic acid-mediated PCL/GLT solution with ALN@MSNs was electrospun. It was found that the healing time was decreased from 12 weeks to four weeks according to the bone repair data from a rat critical-sized cranial defect model [168]. Fibroblast growth factor-2 loaded mesoporous calcium silicate NPs were synthesized and used as fillers of PCL to obtain composite scaffolds with a controlled pore structure. Drug release kinetics, bioactivity, cell proliferation, differentiation, and animal study were performed to confirm the possibility of their application in bone tissue engineering. The presence of mesoporous calcium silicate enabled the incorporation of fibroblast growth factor-2 into the composite scaffolds, and consequently, it was gradually released from the scaffold to facilitate the proliferation and osteogenesis differentiation of human Wharton’s jelly MSCs. The synergic activity of calcium silicate and fibrous growth factor-2 induced the acceleration of new bone formation, which was confirmed via the in vivo femur defect experiments [169]. The modulatory effects of AuNP-loaded MSNs on macrophages and their influence on the osteoblastic lineage cells’ behavior were investigated. The Au-MSNs generated a suitable immune microenvironment by stimulating an anti-inflammatory response and inducing the osteogenic cytokine secretion by macrophages. An improvement of osteogenic differentiation in preosteoblastic MC3T3 cells was confirmed via the increased expression of osteogenic markers, ALP production, and calcium deposition. In an in vivo study, it was also observed that the Au-MSNs fastened bone formation in a critical-sized cranial defect site in rats [170].

Hydrogels based on biopolymers could be suitable substitutes in bone regeneration, but they possessed insufficient mechanical properties and rapid degradation rate. They can be replaced by inorganic/biopolymer hybrid hydrogels prepared via photo-cross-linking of methacrylated GLT and octamethacrylated polyhedral oligomeric silsesquioxane (OMAPOSS) nanocages. Such a hydrogel demonstrated high mechanical strength, better degradation rate, and better biological activity compared to simple hydrogels without POSS. In addition, the attachment, spreading, proliferation, and osteogenesis of MSCs were obviously enhanced in a rat calvarial defect model [171].

NCPs based on a poly(ethylene oxide terephthalate) (PEOT)/poly(butylene terephthalate) (PBT) (PEOT/PBT) copolymer and 2D nanosilicates (SiCs) were tested for the production of 3D scaffolds. It was proven that the PEOT/PBT scaffold promoted calcification and bone bonding ability in vivo. The 2D SiCs induced hMSC osteogenic differentiation in the absence of osteoinductive agents. The stability of PEOT/PBT NCPs in physiological conditions was improved by the addition of SiC because SiC decreased the polymer degradation rate. The bioactive properties of NCPs were also improved. Human MSCs readily proliferated on these scaffolds [172]. Nano-SiC was inserted into a Zn matrix via laser melting to enhance its mechanical properties and was distributed along the Zn grain boundaries, which led to the reduction in Zn grain size from 250 µm to 15 µm with 2 wt% SiC (Zn-2SiC). Nano-SiCs also acted as a reinforcer by virtue of Orowan and dispersion strengthening. Thus, the NCPs exhibited maximum compressive yield strength (121.8 ± 5.3 MPa) and high microhardness (72.24 ± 3.01 HV). The values were increased by 441% and 78%, respectively, compared to pure Zn. After the incorporation of nano-SiC, a more ductile fracture of the NCPs together with suitable biocompatibility and accelerated degradation was indicated [173]. A modified sol–gel processing technique was used to prepare the inorganic–organic composite hydrogel based on PVA and borosilicate gel (BSiC) via gelation and chemical reaction. The hydrogel possessed a uniform single phase with interpenetrating PVA networks. In phosphate-buffered saline (PBS), the PVA–BSiC hybrid-derived scaffolds released ions into the medium and converted to HA. The scaffolds were non-toxic to the rat bone MSCs and promoted their proliferation. The ALP activity of rat bone MSCs and the expression levels of osteogenic-related genes (ALP, OCN, and RUNX-2) increased with an increasing amount of BG [174]. 2D borocarbonitride (BCN) nanosheets were applied as a photothermal agent for osteosarcoma therapy and bone regeneration. Akermanite (Ca_2_MgSi_2_O_7_) bioceramic porous scaffolds were produced by 3D printing; then bifunctional BCN@Akermanite scaffolds were constructed by coating BCN nanosheets on the Akermanite scaffolds. The outstanding photothermal performance of BCN@Akermanite for osteosarcoma therapy was reached due to the BCN strong light absorption. The hydroxyl functional group and boron on BCN nanosheets enhanced bone regeneration, ability of in situ mineralization, fibronectin protein upregulation, and activation of the BMP-2 signaling pathway [175].

Zeolitic imidazolate framework-8 NPS (ZIF-8 NP)-modified catechol (CA)–CS multifunctional hydrogels (CA–CS/Z) were fabricated to stabilize the bone graft environment, ensure blood supply, promote osteogenic differentiation, and accelerate bone reconstruction. Hydrogels demonstrated good rheological properties, suitable mechanical strength, excellent adhesion, biocompatibility, and antimicrobial properties. The hydrogels could promote paracrine of VEGF in rat bone MSCs to ensure blood supply reconstruction in bone defect areas. The ZIF-8 NPs released from the hydrogels could contribute to the regulation of the production and secretion of ALP, type I CLG, and OCN, which promoted the osteogenic differentiation of the rat bone MSCs. In in vivo experiments, it was found that CA–CS/Z significantly hastened the speed and healing of bone repair [176].

The in vitro, ex vivo, and in vivo investigation of bioprinted human bone MSCs encapsulated in a nanoclay-based bioink was conducted to make viable and functional 3D structures. The materials maintained their viability over 21 d in vitro. The 3D scaffolds were seeded with human umbilical vein endothelial cells (HUVECs) and loaded with VEGF implanted ex vivo into a chick chorioallantoic membrane model. The integration and vascularization were shown after 7 d of incubation. BMP-2 absorbed in the scaffolds caused strong mineralization after four weeks (*p* < 0.0001) compared to the drug-free and ALG scaffolds. Bone tissue formation was also confirmed [177]. A non-invasive delivery system based on injectable and self-healing NCP hydrogels for sustained protein release was studied based on laponite (LAP) nanoplatelets, which are able to improve the gelation process through hydrogen bonds with polysaccharide matrices, producing hydrogels with excellent mechanical and rheological behaviors as well as better injectability and self-healing ability. The bond between LAP nanoplatelets and BMP-2 forms stable LAP@BMP-2 complexes that efficiently keep BMP-2 intrinsic bioactivity. As a result, the release period was prolonged for more than four weeks. In addition, boost cell spreading, proliferation activity, and osteogenesis were enhanced in vitro and in vivo in the hydrogels with the LAP@BMP-2 complexes compared to LAP or BMP-2 alone [178]. Bioactive and antimicrobial NCPs were produced using PEGylated polyglycerol sebacate as a NCP base functionalized with LAP SiCs and an antimicrobial peptide (AMP). The elastic modulus and ultimate tensile strength values were between the range of 3.8–4.7 MPa and 1.5–3 MPa, respectively. A significant antimicrobial activity against both Gram-negative (*E. coli*) and Gram-positive (*S. aureus*) bacteria was observed. A > 90% viability of preosteoblast (W-20-17) cells was determined using in vitro cytocompatibility tests, and in vitro differentiation assays exhibited the ability of scaffolds to promote osteogenic differentiation of W-20-17 [179]. Attapulgite is a fibrillar clay mineral with large specific surface area, high viscosity, and high absorption capacity. It was used for the preparation of composite scaffolds consisting of CLG/PCL/attapulgite (CPA) or CLG/PCL using a salt-leaching method. It was observed that the cells exhibited the perfect ability to attach to the CPA scaffolds together with the obvious upregulation of osteoblastic markers such as RUNX-2, osterix, type I CLG, OPN, and OCN. Formation of abundant new bones on the CPA was proven [180].

Polymer composite fibers consisting of PCL, montmorillonite (MMT) nanoclay, and nHA were produced and used for the production of 3D scaffolds to enhance bone growth, cell viability, and proliferation. The produced scaffolds were biocompatible, and the cells were able to adhere and differentiate on them. Higher cell viability, osteogenic differentiation, extracellular matrix, and CLG formation were confirmed using HA–MMT–PCL scaffolds compared to PCL fibers. A better osteogenic differentiation of MSCs was proven by increased intracellular ALP values [181]. The biological properties of Baghdadite (zirconium modified calcium-silicate-based ceramics, Ca_3_ZrSi_2_O_9_) in the form of NPs were investigated. The NPs were nontoxic (MTT assay), and the proliferation of bone marrow derived MSCs was increased after 96 h of culturing. The defected bone was completely regenerated six weeks after the implantation of the NPs [182]. A 3D printing composite consumable containing the PLLA matrix, surface grafted MgO whiskers (gMgOs), and halloysite nanotubes (gHNTs) was prepared to combine the printability of PLLA, the perfect osteogenic activity of gMgOs, and the excellent reinforcement and toughening of gHNTs. The composite scaffolds with large and small pores and honeycomb structure showed increased hydrophilicity, tensile, and compressive properties, together with affinity and osteogenic activity, improved mechanical properties, and promising cell adhesion, proliferation, and migration [183].

### 4.4. Metal-Based Nanomaterials

#### 4.4.1. Magnesium-Based Nanomaterials

Magnesium (Mg) and its alloys have demonstrated suitable biocompatibility and mechanical strength for medical applications. The problem is low Mg corrosion resistance in a physiological environment. nHA coatings can decrease degradation rates and improve the mechanical strength of Mg based implants, and bone healing is enhanced due to their osteoinductivity and osteoconductivity. Conformal nano-to-submicron HA coatings deposited on Mg plates and rods via the transonic particle acceleration process were studied to describe their effects on Mg degradation in vitro. The corrosion resistance of Mg was improved, and the coatings retained 86–90% of the final compressive strength after in vitro immersion in simulated body fluids for six weeks, while uncoated Mg retained only 66% of strength. The degradation of the rods was slower than that of the plates. Better cell adhesion densities were found under indirect contact conditions than under direct contact conditions for the HA coated Mg, which reduced the adhesion densities of bone marrow-derived MSCs on the surface, but increased them under indirect contact. However, it was also found that Mg-based plate and screw devices could be differently degraded, even if they were treated with the same coatings and implanted at the same or similar anatomical parts [184]. Mg-based alloys are known for their rapid degradation and high corrosion rate; for this reason, they are associated with in vivo infections and implant failure. The stability and anti-inflammatory properties of Mg alloys can be improved by their modification with GR NPs. Low quantities of GR (0.18 and 0.50 wt%) were successfully added by spark plasma sintering (SPS) into a Mg alloy with Al (1 wt%) and Cu (0.25 wt%). The degradation rate of Mg-based alloys decreased approx. 4-fold, and the bactericidal activity increased up to 5-fold when 0.18 wt% GR was used. This NCP showed the compressive properties corresponding to those of native cancellous bone (modulus approx. 6 GPa). A high cytocompatibility together with excellent osteogenic properties was proven in in vitro studies with human MSCs [185]. Mg-based NCPs were produced using HA bioceramic nanoreinforcement. The addition of HA increased the yield strength of the alloy matrix and exhibited superior strength and ductility retention post corrosion for 21 days. The presence of HA also improved the hydrophilicity and biocompatibility of the alloy matrix with enhanced corrosion resistance, non-cytotoxicity, and high cell attachment [186]. Khalili et al. [187] studied the effects of hot isostatic pressing and surface anodizing on the properties of an in situ surface modified magnesium matrix NCP with different percentage by weight of HA by stir-centrifugal casting. They wanted to reduce the defects and to replace the Mg/HA surface with a ceramic matrix NCP layer of MgO/HA, which was confirmed by energy dispersive spectroscopy and X-ray diffraction. The 1.8 wt% nHA was homogeneously distributed in the MgO matrix with a well-arranged nanostructure on the surface, which reduced the H2 release and corrosion rate. The lowest thermodynamic tendency for corrosion (−1.345 V) and the corrosion rate of 3.8388 mm/year with the highest protection efficiency of 42.26% in comparison with the as-cast pure magnesium were observed. Thus, it is a promising material for bone implants [187]. HA–MgO NCPs were fabricated with different bioactive compositions. There were changes in the composition of NCPs sintered at 1200 °C because nHA was partially decomposed into β-tricalcium phosphate (β-TCP). The NCP density was in the range from 2.72 ± 0.066 to 3.03 ± 0.093 g/mL, depending on the MgO content (0.0–2.0 wt%). An obvious increase in the mechanical properties of the composite was achieved with increasing MgO amounts. The best mechanical properties were found for the NCP HA–1.0 MgO (e.g., compressive strength 111.20 ± 5 MPa, fracture toughness 136.98 ± 5 MJ/m^3^) compared to the pure n-HA. The NCP surface had a hydrophilic nature, and biocompatibility in terms of cell viability was also reported [188].

MgO NPs were modified with poly(l-lactic acid-co-malic acid) (PLMA) to support the interfacial compatibility in the PLLA scaffold. PLMA has a hydrophilic end (comes from the carboxylic groups of malic acid) and an l-lactic acid chain. Hydrogen bonds were formed between the carboxylic groups and MgO-NPs, and the l-lactic acid chain hydroxyl groups reacted with the PLLA carboxyl groups. Compressive strength and modulus of the fabricated scaffold were significantly enhanced by 47.1% and 237.7%, respectively [189]. NCP materials of PLA/stearic acid-modified MgO (1 wt%) were prepared using blending extrusion. It was found that the long-term degradation of NCPs depended on the filler shape and was accelerated by an increase in the water uptake rate of the PLA matrix. The MgO NCP was affected significantly by the increased hydrophilicity. Thus, the PLA/MgO materials can efficiently regulate the PLA matrix degradation and enhance its bioactivity [190].

**Figure 8:**
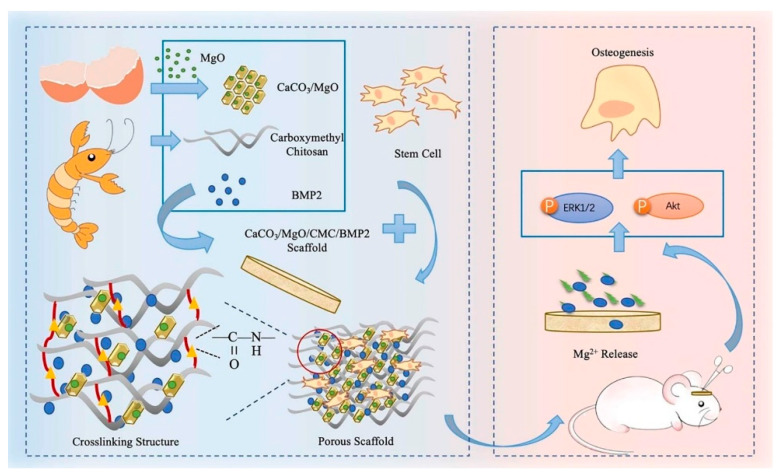
Scheme of the preparation of calcium carbonate/magnesium oxide/carboxymethyl chitosan/bone morphogenetic protein 2 (CaCO_3_/MgO/CMC/BMP2) scaffolds and their applications in vivo. Eggshell micropowder nanoparticles modified using MgO (CaCO_3_/MgO) were prepared in situ and then mixed with bone morphogenetic protein 2 (BMP2) and carboxymethyl chitosan (CMC). A porous scaffold was prepared via chemical crosslinking. The osteoinductivity of this scaffold was characterized at the molecular, cellular, and animal levels. The osteogenic mechanism of the prepared scaffold was related to the release of Mg^2+^ and BMP2, and it could significantly promote seed cell osteogenic differentiation through the ERK1/2 and Akt pathways. Adapted from [193], Elsevier, 2020.

A NCP consisting of PLGA, β-tricalcium phosphate (β-TCP), and Mg(OH)_2_ was designed to promote bone repair through osteoinductive, osteoconductive, and anti-inflammatory abilities. The PLGA/β-TCP/Mg(OH)_2_ NCP increased the bone regeneration rate to fully repair bone defect healing with suppressed inflammatory responses [191]. A polybutylene succinate (PS: 50 wt%), magnesium phosphate (MP: 40 wt%), and wheat protein (WP: 10 wt%) composite (PMWC) scaffold was prepared with interconnected macropores (400 µm to 600 µm) and micropores (10 µm to 20 µm) on the macropore walls. The presence of MP improved the apatite mineralization of the PMWC scaffold in simulated body fluid, and the addition of WP improved the PMWC degradability in PBS compared to the scaffold of the PS/P composite and PS alone. The PMWC scaffold supported the proliferation and differentiation of mouse preosteoblastic cell line (MC3T3-E1) cells, increased new bone formation and ingrowth, and promoted osteogenesis and vascularization, which was confirmed in vivo [192]. The major inorganic component of eggshells is CaCO_3_. Thus, MgO NPs-coated eggshell particles (denoted CaCO_3_/MgO NCPs) were prepared and used for subsequent fabrication of a biomimetic active scaffold based on the chemical crosslinking of the CaCO_3_/MgO NCP, CM–CS, and BMP-2 (see Figure 8). The resulting composites CaCO_3_/MgO/CM–CS/BMP-2 demonstrated a higher modulus and compressive strength than the CM–CS scaffold. The CaCO_3_/MgO/CM-CS/BMP-2 scaffold also showed mineralization ability, osteogenic differentiation potential, and the ability to release Mg^2+^ ions and BMP-2, which could activate the phosphorylation of the ERK1/2 and Akt pathways and promote osteogenesis via the crosstalk of multiple pathways. Outstanding results in bone repair were proven using an in situ rat calvarial defect repair experiment [193].

3D mesoporous biocomposites were designed for dental or bone implant applications based on ZrO_2_–MgO–hBN containing highly porous nanotubes of hexagonal boron nitride (hBN). These were characterized by low density, high strength, and mesoporous interconnected architecture. The materials exhibited interesting properties due to their stability in water, minimum essential medium eagle-α modification (α-MEM), acids, and oils (e.g., suitable proliferation of osteoblast such as MG63 cells or filtration of *E. coli* from water [194]). Magnesium-enriched GO nanoscrolls (MgNPs@GNSs) were designed for the combinational modulation of the inflammatory response. It was proven that GO activates inflammatory M1 macrophages and that Mg^2+^ facilitates the repolarization of M1 macrophages to the pro-healing M2 phenotype. Thus, with the sustained release of Mg^2+^, the MgNPs@GNS nanoplatform can induce synergic type 1 and type 2 inflammatory responses. Mg^2+^ ions decreased the GO internalization and downregulated the nuclear factor κB pathway involved in the inflammatory process. The ordered inflammatory responses stimulated in vitro angiogenesis and osteogenesis through chemotactic, mitogenic, and morphogenic actions, and vascularized bone regeneration was achieved in a rat cranial bone defect model [195].

#### 4.4.2. Titanium-Based Nanomaterials

Titanium (Ti) is a widely used surgical material that is also used for implants. Unfortunately, its bioinertness may be impaired due to poor antibacterial properties. These can be improved, for example, by surface coverage of titanium implants, for example, by a combination of HA and antibacterially active CS, as described by Li et al. [196]. Such a HA–CS composite coating improved cell adhesion and inhibited bacterial growth, and the authors described its applications in orthopedics and dentistry [196].

On the titanium implant Ti-6Al-4V, a hierarchical micro/nano-structured surface supporting the proliferation and osteogenic differentiation of preosteoblast cells (MC3T3-E1) was created by ultrasonic acid etching and anodic oxidation by improving surface hydrophilicity and bioactivity compared to implants with a polished Ti surface, so that the bioactivity and osteogenic properties were improved [197]. In addition, graphdiyne-modified titanium implants have good biocompatibility and osteoinductive capabilities for cell adhesion and differentiation as well as significant antibacterial properties [198]. The Ti6Al4V material coated with a composite composed of nHA and GR showed high corrosion resistance and good biocompatibility [199]. Another way to improve the properties of Ti (Ti–6Al–4V) implants is to cover their surface with GR or CNTs [200]. A HA/TiO_2_/CNT NCP prepared by the hydrothermal method showed good mechanical and physicochemical properties and proved to be suitable for the growth of the human cell line MDA-MB-231 [201].

The AgNP/GO surface-treated titanium Ti–6Al–7Nb implant had significant antibacterial activity against *E. coli* and *S. aureus* and high osteoblast cytocompatibility [202]. In addition, a 0.1 mm thick diamond carbon-coated Ti–6Al–7Nb honeycomb structure exhibiting excellent in vivo properties and high bone growth support has been described by Kawaguchi et al. [203]. The titanium implant Ti16Nb coated with GO, HA, and CLG had increased wettability, which was reflected in increased cell adhesion and fibroblast viability [204].

Titanium coated with a layer of CaO NPs was highly active in vitro against methicillin-resistant *S. aureus* (MRSA) and at the same time, significantly promoted osteogenic differentiation of bone marrow MSCs [205]. nHA coated with polyamide 66 with AgNPs and TiO_2_ NPs showed high antibacterial activity against *E. coli* and *S. aureus* and good biocompatibility. The NCP promoted cell adhesion and cell proliferation of preosteoblasts. Thus, the whole system seems to be suitable for the treatment of osteomyelitis [206].

An electroactive biocomposite of PLGA mixed with gadolinium-doped barium titanate NPs (Gd-BTO NPs) was studied to establish the relationship between surface potential and osteogenic activity. The introduction of the Gd-BTO NPs improved the elastic modulus and was suitable for MRI and X-ray dual imaging. The electrical properties of these NPs (e.g., dielectricity, piezoelectricity, and surface potential electrical characteristics) were effectively improved. The negative surface potential of poled Gd-BTO/PLGA significantly increased cell attachment and osteogenic differentiation, induced intracellular Ca^2+^ ion concentration oscillation, and improved osteogenic differentiation via the calcineurin/NFAT signal pathway [207].

#### 4.4.3. Other Metal-Based Nanomaterials

Highly-crystalline, round-shaped ZnO nanocrystals (ZnO NCs) of 20 nm in diameter were tested in bone implant applications in vitro in a form of stable colloidal solution in ethanol. The NCs were also partially functionalized by anchoring amino-propyl groups to the ZnO surface (ZnO-NH_2_ NCs). The tests of biocompatibility toward preosteoblast cells, promotion of cell proliferation and differentiation, and antimicrobial activity against Gram-positive and -negative bacteria showed that ZnO-NH_2_ NCs are applicable for the treatment of implant-related infectious diseases and could be used as a highly biocompatible and osteoinductive nanoantibiotic agent for bone tissue engineering [208]. The scaffolds with high porosity (approx. 93%) and pore size ranging from 100 to 400 µm based on a poly(d,l-lactide acid) (PDLLA) matrix containing undoped and Cu-, Zn-, and CuZn-doped bioactive glass particles were produced by freeze-drying and salt-leaching methods. Improvements in the elastic moduli were as high as 130%, and the apatite formation on the scaffold surface was induced. The scaffold degradation showed the highest rate in the case of the PDLLA/undoped glass scaffold. Thus, the incorporation of undoped and metal-doped bioactive glasses increased the mechanical strength, promoted the bioactivity, and modified the degradation profile of the scaffolds [209].

A 3D porous iron (Fe) scaffold with skeleton diameter 143 µm, interconnected pores, average pore size 345 µm, porosity > 90%, and yield strength 3.5 MPa was prepared via a template-assisted electrodeposition method. Strontium incorporated octacalcium phosphate (Sr-OCP) was used as a coating of the Fe scaffold skeleton to ensure the biocompatibility. The coating was in the form of nanowhiskers with the mean diameter of 300 nm and the length of 30 µm and decreased the release rate of Fe ions to a level safe for the human body. The cell adhesion and biocompatibility were enhanced [210]. Polymer/phosphate glass/Fe_3_O_4_ MNP (CG/PG/MNP) composite scaffolds were developed using a freeze drying technique. The scaffolds were highly porous containing interconnected pores of the size ranging from 20 to 150 µm. Their swelling and degradation behavior were influenced by the integration of Fe_3_O_4_ MNPs, and they demonstrated slight ferromagnetic properties. The compressive modulus increased with increasing MNP content. Good bioactivity and cytocompatibility were confirmed [211].

Cerium oxide (CeO_2_) NPs have free radical scavenging capabilities. CeO_2_ NPs were incorporated into GLT–ALG scaffolds to obtain NCP scaffolds (GAC) by freeze drying. Various CeO_2_ NP concentrations were used, and their influence on the physicochemical and biological properties of the NCP scaffolds was evaluated. The mechanical properties and bio-mineralization were improved; the swelling and in vitro weight loss of the scaffolds decreased. The ALP activity, RUNX-2, and OCN expression study indicated that the GAC scaffolds support MSC differentiation into osteoblasts. In addition, the GAC are capable of reducing free radicals [212].

### 4.5. Polymers and Other Organic Materials

Due to their unique 3D network structure, high content of water, and functional properties, hydrogels are the next promising candidates for bone tissue engineering. Many studies introduce synthesis methods (e.g., 3D-printing technology) to prepare implanted hydrogel scaffolds with optimal properties. CLG, hyaluronic acid, CS, polyethylene glycol (PEG), and other biocompatible materials are used as injectable hydrogels in minimally invasive surgery. They have adjustable physicochemical properties and can fill irregular shapes of defect sites and release drugs or growth factors via different stimuli (pH, temperature, redox, enzyme, light, magnetic, etc.) [213]. Primary MSCs along with BMP-2 were incorporated into the ALG skeleton. By releasing BMP-2 into the defective bone microenvironment, it caused osteogenic differentiation, leading to the rapid formation of mature bone [214]. Similarly, Jin et al. [215] prepared a NCP composed of PEI-ALG, in which BMP-2 was incorporated. Tests showed that the composite released BMP-2 protein for at least 14 days and that osteogenesis was promoted. At the same time, ALP activity and calcium storage were increased [215]. An engineered implantable scaffold sustainably releasing alendronate (Aln) for osteoporotic bone defects was investigated. Aln was added into 2% CLG solution, and then the mixture was used to obtain CLG–Aln scaffolds. It was found that Aln was released for one month with the average rate of 2.99 µg/d within the first eight days. The CLG and CLG–Aln scaffolds were implanted into 5 mm cranial defects in ovariectomized rats. Better bone regeneration in defect area (11.74 ± 3.82%) after three months was found in the defect implanted with the CLG–Aln scaffolds compared to the CLG scaffold (5.12 ± 1.15%) (*p* < 0.05) [216]. CS/SF/glycerophosphate (GP) composites, to which copper-containing bioactive glass NPs (Cu–BG NPs) were incorporated, were prepared to produce injectable hydrogels for cell-free bone repair. The highly porous Cu–BG/CS/SF/GP gels were well injected, and gelation at physiological temperature and pH was rapid. They could administer Si, Ca, and Cu ions at safe doses. The growth of seeded MC3T3-E1 and HUVECs was supported, and were suitable for osteogenesis and angiogenesis. The Cu–BG/CH/SF/GP gel could fully repair the bone defect, which was obvious thanks to the formation of vascularized bone tissue and mineralized CLG deposition during eight weeks with no cells and/or growth factors being used [217].

An injectable luminescent hydrogel composite composed of PLGA–PEG–PLGA triblock copolymer and NaYF_4_: Yb^3+^, Er^3+^ hollow microtubes was fabricated for noninvasive bone regeneration monitoring. The formed hydrogel had much rougher surface, enhanced mechanical properties and bright luminescence, suitable drug release property for protein drugs, good cellular compatibility, and MSC adhesion. The composite hydrogel with loaded recombinant human bone morphogenetic protein 2 (osteogenic induction factor) was implanted into the tibial defect of rats to evaluate the bone repair. The hydrogel scaffold degradation was completed after four weeks; the repair of the tibial defect was finished after six weeks; and the biological safety was reported [218]. NCPs based on polyurethane (PU), ghee, and propolis were developed using the electrospinning technique. The PU/ghee (817 ± 138.39 nm) and PU/ghee/propolis (576 ± 144.96 nm) NCPs had smaller fiber diameter than the pure PU membrane (890 ± 116.911 nm). The contact angle raised in PU/ghee (122 ± 1°) showed hydrophobic properties, while it was lower in PU/ghee/propolis (55 ± 1°) with hydrophilic behavior. The surface roughness of the NCPs decreased, and their thermal stability was improved. The NCPs were characterized by better blood compatibility with non-hemolytic and non-toxic properties and improved safety to RBCs [219].

Other attractive materials for the fabrication of functional scaffolds are poly(vinylidene fluoride) (PVDF) and poly(vinylidene fluoride-trifluoroethylene) (P(VDF-TrFE) with excellent piezoelectricity and good biocompatibility. Electrospun PVDF and P(VDF-TrFE) scaffolds produced electrical charges during mechanical deformation; thus, they stimulated bone defects and damaged nerve cells repairing. Bone and neural cells were promoted to adhere, proliferate, and differentiate on their surfaces. Additionally, neurite growth along the direction of fiber orientation was enhanced by the aligned PVDF and P(VDF-TrFE) fibrous structure. Small pore sizes prevented the infiltration of bone and neuronal cells into the scaffolds, which led to the formation of a single cell layer on the scaffold surfaces [220]. Magnetoactive 3D porous scaffolds based on PVDF and magnetostrictive particles of CoFe_2_O_4_ were fabricated using a solvent casting method with nylon template structures and three different fiber diameters (60, 80, and 120 µm). The magnetoactive composites had a structure very similar to that of the trabecular bone with pore sizes from 5 to 20 µm due to the crystallization of PVDF in the presence of the NPs, interconnected with bigger pores that were formed after the removal of the nylon templates. The materials crystallized in the PVDF electroactive β-phase, and the proliferation of preosteoblasts was promoted via the application of magnetic stimuli [221].

A new class of citrate-based materials with glycerophosphate salts, β-glycerophosphate disodium (β-GP-Na) and glycerophosphate calcium (GP-Ca) was used for the preparation of the resultant poly(octamethylene citrate glycerophosphate). The tensile strength of POC–GP–Na and POC–GP–Ca was approx. 28.2 ± 2.44 MPa and 22.76 ± 1.06 MPa, respectively. The initial modulus ranged from 5.28 ± 0.56 MPa to 256.44 ± 22.88 MPa. POC–GP–Ca exhibited higher cytocompatibility and the corresponding composite POC–GP–Ca/HA showed enhanced osteogenic differentiation of human MSCs in vitro compared to POC–GP–Na/HA and POC/HA. The better in vivo performance of the POC–GP-Na/HA scaffolds was confirmed in a rabbit femoral condyle defect model [222].

## 5. Critical View and Perspectives

It is important to note that the diseases discussed above are caused by systemic imbalances (in the case of osteoporosis) and whole-joint degenerative disease (in the case of osteoarthritis) involving all joint tissues including articular cartilage, subchondral bone, infrapatellar fat pad, synovium, ligaments/tendons, and menisci. Therefore, joint replacement or improved/facilitated healing (cartilage/bone tissue regeneration) with nanocomposites is not really a cure, but only an attempt to correct the consequences of the disease, which does not address the underlying cause of the disease. Therapeutic treatment with “classical” drugs focuses primarily on cartilage damage as the primary lesion without taking into account other joint tissues and their impact on the pathology of joint diseases. Unfortunately, as history and the present show, these approaches are not entirely successful, and managing arthritis is still a major challenge.

Currently, there are no approved drugs that could vigorously alter the course of degenerative joint diseases and cause long-term, clinically significant benefits. For a therapeutic intervention to be effective, disease-modifying drugs able to modulate many different cell types present in the joints are needed to be developed, so that many pathophysiological processes can be corrected and a “global” therapy can actually be formed [6,10,11,12,13,16,21,22,23,24,27,223]. As outlined, individual and combinatorial approaches to the treatment exist or are being developed, and many materials are being prepared to replace degenerate tissues using 3D printing and bioscaffolds. Similarly, transport systems have been developed for a number of chemical and biological therapeutics.

Since 2010, when the private sector became interested in tissue engineering again after the crisis, the first commercial products focused on soft tissue replacements such as artificial skin applicable as a coating for burns appeared on the market. Other products can be found in the cosmetics industry. It is also positive that the current regulatory framework, especially in the U.S., facilitates the faster commercialization of tissue engineering products [224,225]. In the European Union, their introduction into the market is more complicated; however, the tissue engineering market in the EU is estimated to grow, especially for companies operating in the 3D bioprinting market, thanks to growing government support and growing demand for cosmetic surgery. Implants suitable for teeth and jawbones and osteotransplants for bone surgery applications can be found on the market. Osteoinductive products containing collagen, growth factors, and human cells that induce bone growth and have the potential to strengthen damaged or weakened bones or create new bones have been marketed [226,227]. Unfortunately, despite all the expectations, promising advances, and an incredible increase in professional studies, tissue engineering based on nanocomposite scaffolds faces numerous challenges (i.e., translating all expectations and plans into reality is a complicated task). Although nanotechnology, through nanoparticle engineering, is expected to have the huge potential to solve all problems, there are still obstacles to the clinical and widespread use of nanocomposites due to the inability of artificial solid materials to mimic the natural properties of tissues.

## 6. Conclusions

Biomedical applications of nanomaterials have a significantly increasing trend. In addition to their use as drug carriers and diagnostics, in recent years, various nanocomposites have been used as scaffolds in tissue engineering to help to prepare biomimetic replacements to repair damaged tissues and organs. As the number of degenerative diseases of the musculoskeletal system increases and the traditional therapy has reached its limits, the use of these implants in orthopedics and rheumatology, where various nanocomposites for the healing of cartilages in joints and damaged bones are used or investigated, is not surprising. Currently, the most preferred are polymeric materials, hydroxyapatite, and carbon- or silica-based materials. These are variously functionalized to facilitate the proliferation of healthy cells in the affected tissues, increase biocompatibility, and reduce toxicity. Functionalization can also serve to encapsulate drugs (i.e., the implant can also have antibacterial, anti-inflammatory, or anti-cancer properties). On the other hand, many nanomaterials are known to cause inflammatory processes and tissue damage. These negative phenomena can be eliminated precisely by functionalization, careful testing, and uncompromising selection of the most suitable, stable, and biocompatible nanomaterials. Thus, the advanced design of nanostructured scaffolds has the ambition to provide synthetic nanocomposites with more advantageous properties for native tissue, which in turn will lead to increased and accelerated healing and the formation of functional regenerated tissues.

## Figures and Tables

**Table 1:** Summary of composite nanomaterials developed for cartilage healing and regeneration.

Scaffolds	Fillers	Tested Cell Cultures	In Vitro Tests	In Vivo Tests	Preparation	Effect	Ref.
Collagen, chitosan	Micro graphene oxide, BMP-2 (1:1)	Chondrocytes	SEM, immunofluorescence	Rats, knee, femur cartilages	3D printed	Enhanced chondrocyte proliferation	[83]
Polycaprolactone	Graphene nanoplatelets, polycarboxylate modified graphene nanoplatelets 0.5, 5, 10 wt%	Human chondrocytes knee, hip	Cytotoxicity, cell proliferation	-	Injection molding process to make filaments in form of sticks, 3D printing	Improved mechanical properties, support of proliferation of chondrocytes	[84]
Acellular cartilage extracellular matrix, distal femoral condyle of market-weight pigs	Graphene oxide 0, 1, 2, 4, 6 mg/mL	Chondrocytes	Cell viability, adhesion, and proliferation, chondrogenesis	Implantation in rats, cartilage defect model in rabbits and histological evaluation	-	Improvement of internal structure and mechanical properties	[85]
Sericin	Reduced graphene oxide in ratio 10:1, 50:1, 100:1	Mesenchymal stem cells derived from bone marrow of humans	Mesenchymal stem cells differentiation, growth, adhesion	-	-	Increased levels of collagen and glycosaminoglycan, chondrogenic differentiation stimulation	[86]
Gelatin, methacrylate polyethylene (glycol) diacrylate	Graphene oxide	Primary human bone marrow mesenchymal stem cells	Mesenchymal stem cells proliferation, chondrogenic differentiation, collagen II secretion, glycosaminoglycan synthesis, total collagen levels, RT-PCR	-	3D printed scaffolds	Favorable mechanical properties, biocompatibility, increased collagen, glycosaminoglycan, protein levels; chondrogenic differentiation of mesenchymal stem cells	[87]
Chitosan, gelatin, anionic non-sulfated glycosaminoglycan	Graphene 0, 0.024, 0.06, 1%	Bone marrow mesenchymal stem cells	-	-	Bioink, 3D-printing	Enhanced water absorption, porosity, compression modulus, cytocompatibility, cell growth, higher cells proliferation survival	[80]
Poly(ε-caprolactone)	Graphene nanopowders 1, 3, 5, 10 wt%	Mouse bone marrow mesenchymal stem cells	Cell culture studies, MTT Assay, Live/Dead^®^ assays, glycosaminoglycan formation, cell attachment and morphology	-	Printing ink, 3D-printing, robocasting method	Highest cell viability rates of cells seeded onto composite scaffolds, cells proliferated well, attached to scaffold surfaces	[88]
Polycaprolactone	Graphene and single-wall carbon nanotubes, 0.5% and 1.0% poly-l-lysine coated	Mesenchymal stem cells	Mesenchymal stem cells cell adhesion, proliferation, and chondrogenic differentiation	-	Electrospinning, microfibrous scaffolds	Improved mechanical properties, more homogenous fiber morphology, surface properties, good cytocompatibility	[79]
α-Chitin, poly(caprolactone)	Chondroitin sulfate, transforming growth factor-β encapsulation	Adipose derived stem cell from inguinal fat pads of female New Zealand albino rabbit	Cell viability, attachment, and proliferation study, chondrogenic differentiation and analysis of a murine rheumatoid arthritis model	-	Lyophilization technique	Prolonged release of TGF-β achieved, macroporous, extremely porous structure, enhanced cell attachment, proliferation, differentiation	[81]
-	Graphene oxide granules	Umbilical cord mesenchymal stem cells	-	Male New Zealand white rabbits: expression levels of nitric oxide, interleukin-6, tumor necrosis factor-α, glycosaminoglycan, collagen-II in serum and articular fluid	Mixing	Reduction in inflammatory level, improve of level of biochemical environment in articular cavity, promotion of cartilage repair	[89]
2% chitosan	0, 0.1, 0.2, 0.3 (*w*/*v*) % suspensions of graphene oxide in deionized water	Human articular chondrocytes	MTT assay	-	Ultra-sonication process	Improvement of physical, mechanical properties, increased proliferation of human articular chondrocytes	[90]
Poly(lactide-co-glycolide acid)	Graphene oxide	Bone marrow mesenchymal stem cells	Rabbit bone marrow mesenchymal stem cells	Rabbit supraspinatus tendon repair model	Electrospining	Accelerated proliferation and osteogenic differentiation, promoted healing, increased bone and cartilage generation, improved collagen arrangement	[91]
Collagen-I, genipin	Carbon dots	Bone marrow derived stem cells	Chondrocyte differentiation medium, intracellular ROS production, Cell Counting Kit (CCK)-8 assay, cell viability	Articular cartilage intracellular ROS production	Mixing	ROS production by photodynamic therapy, enhanced cartilage regeneration, chondrogenic differentiation, increased stiffness, reduced degradation	[92]
Collagen-II, chitosan, poly(lactic-co-glycolic acid)	-	Rabbit chondrocytes labelled with magnetic Iron oxide nanoparticles, TANBead^®^ USPIO-101 (Amine group, Taiwan Advanced Nanotech Inc., Taipei, Taiwan)	Cell proliferation assay reagent WST-1, cell viability, cytotoxicity, relative proliferation activity	New Zealand White rabbits: levels of chondrogenetic marker genes including Sox-9, aggrecan, collagen-II	Mixing	Incorporation of chondrocytes into cartilage by magnetic force	[93]
Chitosan, collagen-I	Bioactive glass nanoparticles	Human osteosarcoma cell culture (SAOS) and kidney cells line of human embryo (HEK 293T)	The cytotoxicity and cell viability of hydrogels, MTT, Live/Dead^®^ assays	-	Mixing	Improvement of physicochemical, morphological and rheological properties	[94]
2-Hydroxypropyltrimethyl ammonium chloride chitosan, polyvinyl alcohol	Nano-hydroxyapatite, sodium citrate dihydrate	Mouse preosteoblast cells MC3T3-E1	Tests of cell viability and proliferation	-	Freezing/thawing technique and immersing process	Improvement of mechanical and tribological properties, biological compatibility	[95]
Polyvinyl alcohol, polyvinyl pyrrolidone	Stick-like TiO_2_ nanostructures	Human osteosarcoma (HOS; MG-63) cell line	Osteoblast adhesion and proliferation	-	Sol–gel method	Excellent antibacterial efficiency, well cell adhesion and proliferations, bone formation improved	[96]
Glycol, chitosan	Nano-hydroxyapatite	Human sarcoma cell line culture, kidney cell line of a human embryo culture (HEK293T cells) and human bone marrow mesenchymal stem cells (HBMS)	MTT assay, Live/Dead^®^ assays	-	solvent cast and evaporation	Potential bone-related biomedical applications	[97]
Chitosan, β-glycerophosphate disodium salt, gelatin	Bioactive glass nanoparticles	Rat bone marrow mesenchymal stem cells	Cytocompatibility of the hydrogels	Injecting hydrogels into dorsum of Swiss rats	Sol gel method	27% increase in cell viability	[98]
Alginate, polyvinyl alcohol	Chondroitin sulfate loaded zein nanoparticles	Chondrocytes	Degradation studies, chondrocyte culture, Live/Dead^®^ assays, MTS assay, RT-PCR, western blotting	-	Constant stirring and ultrasonication	Chondrocyte improvement, biomimetic matrices upregulating early chondrogenic marker gene (Sox-9) and differentiated genes specific for hyaline cartilage	[99]
Cellulose nanocrystal/dextran hydrogels	Kartogenin and ultrasmall superparamagnetic iron-oxide	Bone marrow-derived mesenchymal stem cells	CCK-8 assay, Live/Dead^®^ assays, gene expression levels	Rabbit articular cartilage	-	Mechanical strength, kartogenin long-term release, support of hyaline cartilage regeneration	[100]

**Table 2:** Summary of composite materials containing nano-hydroxyapatite as basic filler (only any other fillers are listed).

Matrix	Filler	Tested Cells	In Vitro	In Vivo	Ref.
Carboxymethyl chitosan	Sodium alginate	-	MTT assay, life/dead assays	-	[108]
Poly(d-lactic acid)		Bone mesenchymal stem cells	Proliferation assay, live/dead assays, osteogenic differentiation	-	[109]
Poly(l-lactic acid)	Mesoporous silica Santa Barbara Amorphous-15	MG63 osteoblast cells	MTT assay, cell proliferation, osteogenic differentiation,	-	[110]
Poly (lactide-co-propylene glycol-co-lactide) dimethacrylate	Hydroxyethyl methacrylate	Long-term release BMP-2	Biocompatibility in rat mesenchymal stem cells, live/dead assays, proliferation cell, osteogenesis, gene expressions of osteogenesis-related markers	Rabbit femoral condyle defect animal model, micro-CT, histological observations	[111]
Poly(lactic-co-glycolic acid)	Poly(γ-benzyl-l-glutamate)	Mouse preosteoblast cells MC3T3-E1	Cell culture, viability and morphology assay using mouse preosteoblast cells, MTT assay, ALP assay	Repair of rabbit radius defect, X-ray, micro-CT tests	[112]
Acrylated epoxidized soybean oil, polyethylene glycol diacrylate, phenylbis(2,4,6-trimethylbenzoyl)phosphine oxide		-	-	-	[113]
Chitosan, poly(methyl vinyl ether-alt-maleic anhydride		SD rat bone marrow mesenchymal stem cells	Biocompatibility, viability of cells	-	[114]
hydroxyl-capped poly(lactide), carboxyl-capped aniline pentamer, poly(lactide-co-glycolide)	l-Lactic acid oligomer	Mouse preosteoblast cells MC3T3-E1	MC3T3-E1 cell proliferation activity with and without electrical stimulation, MTT assays	Intramuscular implantation into rabbits dorsal muscles, implantation for repair of radius defects in rabbits and of tibia defects in sheep	[115]
Poly(thioketal urethane)		-	-	Femoral defects in New Zealand White rabbits	[116]
Poly(butylene-adipate-co-terephthalate)	Graphene nanoribbons	-	-	Implantation into critical tibia defects in rats, radiography analysis, tomography, bone remodeling, biomechanical properties	[117]
-	Gold nanoparticles	Human bone marrow-derived mesenchymal stem cells	Cell viability, proliferation by CCK-8 assay, Alizarin red S staining, RT-PCR, western blotting	-	[118]
Alginate	Chitooligosaccharide coated silver nanoparticles	MG-63 cells	Antimicrobial testing, MTT assay, cell viability and proliferation, Hoechst 33342 staining assay	-	[119]
Alginate	Zinc	Murine osteoblastic mycoplasm-free cell line	Cytocompatibility assay, cell viability, cytotoxicity assays	Wistar rats: implantation into critical-sized calvarial defects, histological preparation, histomorphometric evaluation, degradation, bioavailability	[120]
-	Zirconia	Mouse osteoblast precursor cell line	Cell cytocompatibility, adhesion, proliferation and differentiation	-	[121]
-	Magnetite	Mouse preosteoblast cells MC3T3-E1	Cell proliferation and morphology	Female SD rats: protein corona formation and determination	[122]
Bacterial cellulose	Magnetite	Mouse fibroblast L929 cells	Cell cytocompatibility on MC3T3-E1, proliferation	-	[123]
Dextran-grafted iron oxide		Human-derived osteoblast-like cells	Cell cytocompatibility, gene expression, RNA isolation and reverse transcription, RT-PCR	-	[125]

**Table 3:** Summary of composite materials containing nanocarbons developed for bone healing and regeneration.

Matrix	Filler	Tested Cells	In Vitro	In Vivo	Ref.
Poly-(l-lactic acid)	Carbon nanotubes	Human osteosarcoma MG63 osteoblast cells	Cell morphology, viability, proliferation	-	[127]
Poly(l-lactic acid)	Graphene nanoribbons, nano-hydroxyapatite	-	*Allium cepa* test, hemolysis	Female Wistar Rats: surgical defects in tibias, comet assay, bone regeneration	[128]
Collagen	Carbon nanotubes, hydroxyapatite	Rat bone mesenchymal stem cells	Cell morphology, viability, proliferation, BMP-2 level, insulin-like growth factor 1 receptor	Female SD rats: X-ray, mason staining and toxicology experiments	[129]
Arginine-glycine-aspartic acid/BMP-2 peptides, poly-(l-lactic acid)	Carbon nanotubes with carboxyl and amino groups	Mouse preosteoblast cells MC3T3-E1	Cell adhesion, proliferation, differentiation, mineralization	-	[130]
Oligo(poly(ethylene glycol)fumarate), poly(ethylene glycol)acrylate	Carbon nanotubes, black phosphorus	MC3T3 preosteoblast cells	Cell adhesion, proliferation, osteogenic differentiation, electric stimulation enhanced osteogenesis	-	[131]
-	Multi-walled carbon nanotubes, nano- hydroxyapatite	Human adipose-derived mesenchymal stem cells	Cell adhesion, attachment, strength, proliferation, osteogenic differentiation, RT-PCR, DNA, ALP assay, total protein analyses	New Zealand white rabbits: dorsal musculature, histological examinations, collagen-I immunostaining analysis, bone-mineral content, macrophage infiltration	[132]
Polycaprolactone	Multi-walled carbon nanotubes	UMR-106 cells	Mitochondrial activity of osteoblasts, MTT assay	Male Wistar rats: immunohistochemistry, extraction of bone proteins	[133]
Polycaprolactone	Multi-walled carbon nanotubes, nano-hydroxyapatite	Human adipose-derived stromal/stem cells	Cell proliferation, osteogenic differentiation, mineralization, Alamar blue assay, ALP assay, amount of collagen and osteocalcin, Alizarin red S staining	-	[134]
Polycaprolactone	Multi-walled carbon nanotubes, eggshell	Bone marrow mesenchymal stem cells	Adhesion and proliferation of osteoblasts	-	[135]
Polyion complex (sodium *p*-styrenesulfonate, 3-(methacryloylamino)propyl-trimethylammonium chloride)	Multi-walled carbon nanotubes	Rat bone marrow-derived mesenchymal stem cells	Biocompatibility, osteogenic differentiation, viability and morphology, Alizarin red S staining, gene expression analysis	SD rats: calvarial defect, micro-CT, histological and immunological staining	[136]
-	Nitrogen-doped multiwalled carbon nanotubes, cellulose, nano-hydroxyapatite	SPF Spragu–Dawley rats mesenchymal stem cells	Cellular attachment, proliferation, viability, mineralization, ALP assay, osteogenic gene expressions	Distal femoral condyle critical size defect in rabbit, bone regeneration and micro-CT analysis, histological analysis	[138]
Chitosan	Graphene oxide	Human adipose derived stromal/stem cells	Cell viability, proliferation, MTT, cytotoxicity, Live/Dead^®^ assays, distribution and morphology, osteogenic differentiation	Mouse models with a calvaria bone defect, Osx osteogenic marker evaluation	[139]
-	Hydroxyapatite, hydrophilic graphene	Mouse preosteoblast cells MC3T3-E1	MTS assay, cell adhesion, proliferation	-	[140]
Carrageenan, acrylic acid	Graphene, hydroxyapatite	Mouse preosteoblast cells MC3T3-E1	Cell viability and proliferation using neutral red dye assay	-	[103]
Poly-ε-caprolactone	Graphene	Mouse preosteoblast cells MC3T3-E1	Cell adhesion and growth behaviors, MTT assay, ALP assay	-	[141]
Polyether ether ketone	Graphene nanosheets	Bone marrow mesenchymal stem cells	Antibacterial screening, cytocompatibility, bone regeneration, Live/Dead^®^ assays, MTT assay, tumor inhibition	Laser treatment on a nude mouse, P/G10 and P/G10-HA tumor growth inhibition	[142]
Polyether ether ketone	Graphene oxide	Bone marrow stromal stem cells	Cell adhesion, cytotoxicity	-	[143]
Polyether ether ketone	Graphene oxide, nano-hydroxyapatite	Mouse preosteoblast cells MC3T3-E1	Cell morphology, proliferation	-	[144]
-	Graphene oxide, nano-hydroxyapatite	Mouse preosteoblast cells MC3T3-E1, stem cells derived from human dental pulp	Bioactivity assay, cytotoxicity analysis, MTT assay	-	[145]
Chitosan	Graphene oxide, nano-hydroxyapatite	Mouse preosteoblast cells MC3T3-E1	Degradation behavior, biomineralization study, Live/Dead^®^ assays	SD rats: histological assessment, bone specific proteins, osteogenesis gene expression	[146]
Spermine based polyurethane-urea	Graphene oxide, 2D rod-like nano-hydroxyapatite,	MG63 osteoblast cells	Cell viability and proliferation, RT-PCR, cell osteogenesis induction, antibacterial study	Mature male SD rats: tibial model, histological assessment	[147]
Shape-memory polyurethane	Isocyanate modified graphene oxide	-	-	-	[148]
-	Graphene nanoribbons, nano-hydroxyapatite	-	-	Wistar rats: histological, biochemical, and radiographic analyses	[149]
Chitosan, quaternized chitosan	Graphene oxide, strontium-substituted hydroxyapatite	Bone marrow stromal cells	Antibacterial test, cytocompatibility using CCK-8 assay, ALP activity	Male SD rats	[150]
Collagen	Strontium-graphene oxide	Human adipose-derived stem cells, human umbilical vein endothelial cell	Cytotoxicity assay, viability, morphology, adhesion, osteogenic differentiation, RT-PCR, western blot, angiogenic effects	Male rats: micro-CT analysis, micro-CT angiography, calvarial undecalcified sections, calvarial decalcified sections	[151]
Collagen-I	Graphene oxide	Rat bone marrow mesenchymal stem cells	Bioactivity, biodegradation, cytocompatibility	Male Wistar rats: biodegradation; male SD rats: craniofacial bone defect study	[152]
Poly(lactic-co-glycolic acid), β-tricalcium phosphate	Graphene oxide, BMP-2	Rat bone marrow-derived mesenchymal stem cell	Peptide release and scaffold degradation, cell viability, adhesion, morphology, osteogenic differentiation	Male Wistar rats: micro-CT and histological evaluation	[153]
Silk fibroin	Graphene oxide, BMP-2	Bone marrow mesenchymal stem cells	Degradation, cell proliferation, adhesion, Live/Dead^®^ assays, osteogenic differentiation, RT-PCR	Male SD rats: calvarial bone defect implantation, micro-CT measurement, histological evaluation	[154]
Silk fibroin, hydroxyapatite	Polyethylenimine functionalized graphene oxide, miR-214 inhibitor	Mouse preosteoblast cells MC3T3-E1, bone marrow mesenchymal stem cells	Cytotoxicity, CCK-8 assay, osteoblast cell proliferation and differentiation, degradation	Calvarial bone-defect model in rats, micro-CT, implantation subcutaneously on back of nude mice	[155]
Alginate, gelatin	Graphene oxide, human mesenchymal stem cells-laden	Human mesenchymal stem cells	Live/Dead^®^ assays, cell morphology, DNA content, ALP activity, osteogenic-related gene expression, micro-CT, histology staining	-	[156]
-	Graphene oxide, mesoporous bioactive glasses	Rat bone marrow mesenchymal stem cells	Cells proliferation, adhesion, ALP assay, immunofluorescence evaluation, Alizarin red S staining, OCN immunofluorescence assay, osteogenic-related gene expression	Male SD rats: cranial bone defect model, micro-CT, microfil perfusion in bone defect, sequential fluorescent labeling in bone defect, newly bone formation and mineralization analysis, immunohistochemical analysis	[157]
-	Fe_3_O_4_/graphene oxide, α-tricalcium, phosphate/calcium sulfate, hydroxypropyl methylcellulose	Rat bone marrow derived mesenchymal stem cells	Antitumor effect of hyperthermia, cell viability via CCK-8 assay, Live/Dead^®^ assays proliferation and osteogenic activity	Nude mice, antitumor efficiency, osteogenesis,	[158]
-	Reduced graphene oxide, nano-hydroxyapatite	Rat bone marrow mesenchymal stem cells	Cell morphology, adhesion, proliferation, Alizarin red S staining, ALP assay, osteopontin expression	Male SD rats: segmental diaphyseal bone defect on, micro-CT, histological changes, bone volume fraction, trabecular thickness and separation	[47]
-	Reduced graphene oxide, zinc-doped hydroxyapatite	Mesenchymal stem cell	Osteogenic differentiation, Alizarin red S staining, osteogenic marker expression, runt-related transcription factor 2, ALP assay, levels of collagen-1, BMP-2, osteocalcin, osteopontin, antimicrobial activity	Rats and mice, a rat femur osteotomy model, micro-CT, calcein evaluation, angiogenesis	[159]
Shape memory polyurethane	Reduced graphene oxide, hydroxyapatite, arginyl-glycyl-aspartic acid	Rabbit bone mesenchymal stem cells	Adhesion	-	[160]
Gelatin	Reduced graphene oxide, methyl vanillate	Bone marrow stromal cells	ALP assay, Alizarin red S staining, immunofluorescence staining, gene expression, endocytosis of bone marrow stromal cells, osteogenic effect	-	[161]
Polyvinyl alcohol	Carbon nanoparticles nano-demineralized bone	MG63 osteoblast cells	Biomineralization, antimicrobial screening, cell viability via MTT assay	-	[162]
Ascorbic acid, polyethylenimine	Carbon dots, miR-2861,	Bone marrow stromal cells	ALP assay, Alizarin red S staining, RT-PCR, fluorescent images, cytotoxicity	Male Wistar rats: intra-tibial injection for bone formation and calvarial bone defect for bone repair on fluorescent images and cytotoxicity, osteogenic effects, bone regeneration, biocompatibility	[163]
Gelatin, hydroxyapatite	Zinc-passivated carbon dots	Rat bone marrow stromal cells	Cytotoxicity and proliferation via MTS assay, intracellular ROS detection, ALP assay, Alizarin red S staining	Male SD rats: calvarial bone defect, radiographic and histological analysis	[164]
Silk fibroin	Carbon nanocages, BMP-2	Rat bone marrow mesenchymal stem cells	ALP assay, cell adhesion, cytotoxicity, RT-PCR	Rats, micro-CT, volume percentage of new bone formation	[165]
-	Carbon nanofiber, gold nanoparticles	MG63 osteoblast cells	MTT and lactate dehydrogenase toxicity assays, cell attachment, morphology	-	[166]
Polyether ether ketone	Carbon nanotubes and fibers hydroxyapatite	Rat bone marrow and raw cell culture	Cytotoxicity and cell viability, MTT assay, ALP assay, telomerase repeated amplification protocol assay	-	[167]

## Data Availability

Data is contained within the article.

## Abbreviations

2D (two-dimensional); 3D (three-dimensional); α-MEM (minimum essential medium eagle-α); α-TCP (α-tricalcium phosphate); β-GP-Na (β-glycerophosphate disodium); β-TCP (β-tricalcium phosphate); ACM (acellular cartilage extracellular matrix); AFM (atomic force microscopy); AGA (arginyl-glycyl-aspartic acid); ALG (alginate); Aln (alendronate); ALP (alkaline phosphatase); AMP (antimicrobial peptide); ATF4 (activating transcription factor 4); AuNPs (gold nanoparticles); BC (bacterial cellulose); BCN (borocarbonitrides); BMP-2 (bone morphogenetic protein-2); BMSCs (bone marrow stromal cells); BP (black phosphorus); BSiC (borosilicate gel); CA (catechol); CCK-8 (Cell Counting Kit-8); CDs (carbon dots); ChS (chondroitin sulfate); CLG (collagen); CM (carboxymethyl); CNCs (carbon nanocages); CNF (carbon nanofiber); CNTs (carbon nanotubes); CNT-PEGA (poly(ethylene glycol)acrylate); –COOH (carboxylic groups); COS (chitooligosaccharide); CS (chitosan); DLP (digital light processing); EDS (energy-dispersive X-ray spectroscopy); Gd-BTO (gadolinium-doped barium titanate); GLT (gelatin); GLY (glycol); GNRs (graphene nanoribbons); GO (graphene oxide); GP-Ca (glycerophosphate calcium); GR (graphene); HA (hydroxyapatite); hBN (hexagonal boron nitride); HEMA (hydroxyethyl methacrylate); hGR (hydrophilic GR); HRT (hormone replacement therapy); HUVECs (human umbilical vein endothelial cells); KGN (kartogenin); LAP (laponite); miRNA (microRNA); MMT (montmorillonite); MRSA (methicillin-resistant *S. aureus*); MSCs (mesenchymal stem cells); MSN (mesoporous silicate nanoparticle); MV (methyl vanillate); MWCNTs (multi-walled carbon nanotubes); NCPs (nanocomposites); NCs (nanocrystals); nHA (nano-hydroxyapatite); NPs (nanoparticles); –O– (ether groups); OA (osteoarthritis); OCN (osteocalcin); –OH (hydroxylic groups); OPN (osteopontin); PBAT (poly(butylene adipate-co-terephthalate)); PBLG (poly(γ-benzyl-l-glutamate)); PBT (poly(butylene terephthalate)); PCL (polycaprolactone); PDLLA (poly(d,l-lactide acid)); PDT (photodynamic therapy); PEEK (polyether ether ketone); PEG (polyethylene glycol); PEGDA (poly(ethylene glycol)diacrylate); PEI (polyethylenimine); PEOT (poly(ethylene oxide terephthalate)); PIC (polyion complex); PLGA (poly(d,l-lactic-co-glycolic acid)); PLLA (poly(l-lactic acid)); PLMA (poly(l-lactic acid-co-malic acid)); PMMA (polymethyl methacrylate); P(MVE-alt-MA (poly(methyl vinyl ether-alt-maleic anhydride)); POC-GP (poly(octamethylene citrate glycerophosphate)); PS (polybutylene succinate); PTKUR (poly(thioketal urethane)); PU (polyurethane); PUU (polyurethane-urea); PVA (polyvinyl alcohol); PVDF (poly(vinylidene fluoride)); P(VDF-TrFE (poly(vinylidene fluoride-trifluoroethylene)); qCS (quaternized chitosan); RA (rheumatoid arthritis); rGO (graphene oxide); ROS (reactive oxygen species); RUNX-2 (runt-related transcription factor 2); RT-PCR (real-time polymerase chain reaction); SASP (senescence-associated secretory phenotype); SBA15 (Santa Barbara Amorphous-15); SD rats (Sprague–Dawley rats); SEM (scanning electron microscope); SF (silk fibroin); SiCs (nanosilicates); SMPs (shape memory polymers); SMPU (shape memory polyurethane); SPIO (superparamagnetic iron-oxide); SPS (spark plasma sintering); Sr-OCP (strontium incorporated octacalcium phosphate); TGF-β (transforming growth factor-β); VEGF (vascular endothelial growth factor); WP (wheat protein); ZIF-8 (zeolitic imidazolate framework-8 NPS).
